# Supplementary material for: Difluoromethylornithine rebalances aberrant polyamine ratios in Snyder–Robinson syndrome
Source: EMBO Mol Med. 2023 Sep 13;15(11):e17833. doi: 10.15252/emmm.202317833 (PMC10630878; doi:10.15252/emmm.202317833)
Supplement: Supplementary file 1 — Expanded View Figures PDF [file EMMM-15-e17833-s006.pdf]

Expanded View Figures

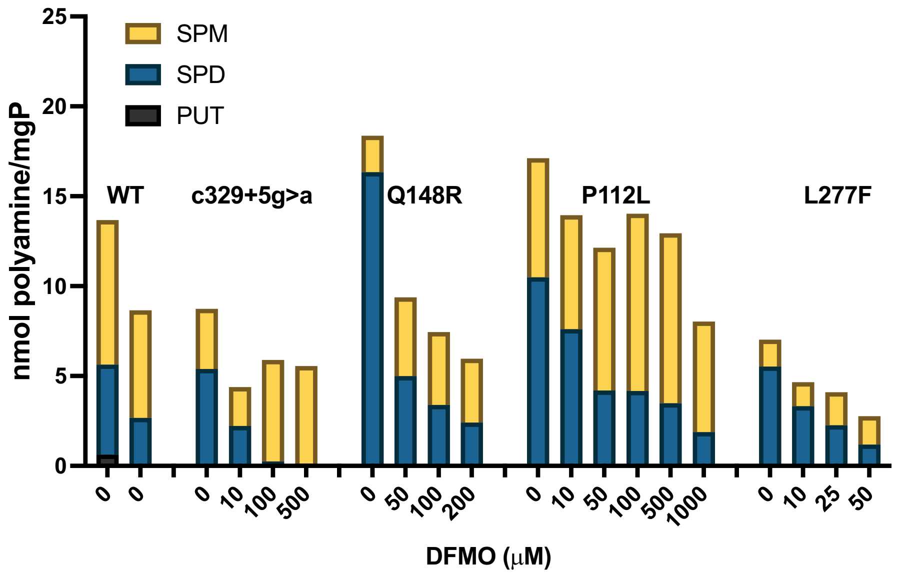

Figure EV1. Effect of DFMO on SPD and SPM pools in SRS patient fibroblast cell lines.

Fibroblasts with hypomorphic mutations of SMS (c.329+5, Q148R, P112L, and L277F) were treated for 96 h with increasing doses of DFMO and analyzed by HPLC for intracellular polyamine levels. A dose-dependent reduction in SPD is observed concurrent with increased SPM concentrations. Data represent the means of duplicate determinations and is presented as nmol polyamine per mg total protein in the lysate. Stacking of individual polyamine levels allows visualization of total polyamine concentration at baseline and with treatment. Cell lines from two different WT donors are included for comparison.

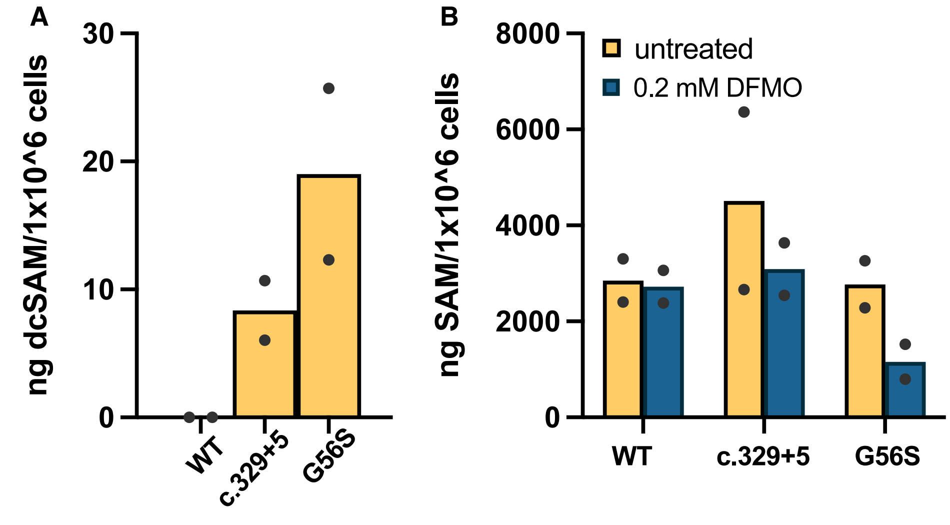

Figure EV2. Concentrations of dcSAM and SAM in lymphoblastoid cell lines.

A Baseline concentrations of dcSAM in SRS lymphoblasts compared to wildtype (WT) were determined by LCMS/MS. Levels were below the limit of detection in WT cells but were elevated in SRS cells, with the highest accumulation in cells from the more severely affected SMS^{G56S} variant.

B WT and SRS lymphoblasts were treated 96 h with 0.2 mM DFMO. SAM levels were quantified by LCMS/MS and normalized by cell number.

Data information: values are plotted from independent experiments with column height indicating the means.

© 2023 The Authors

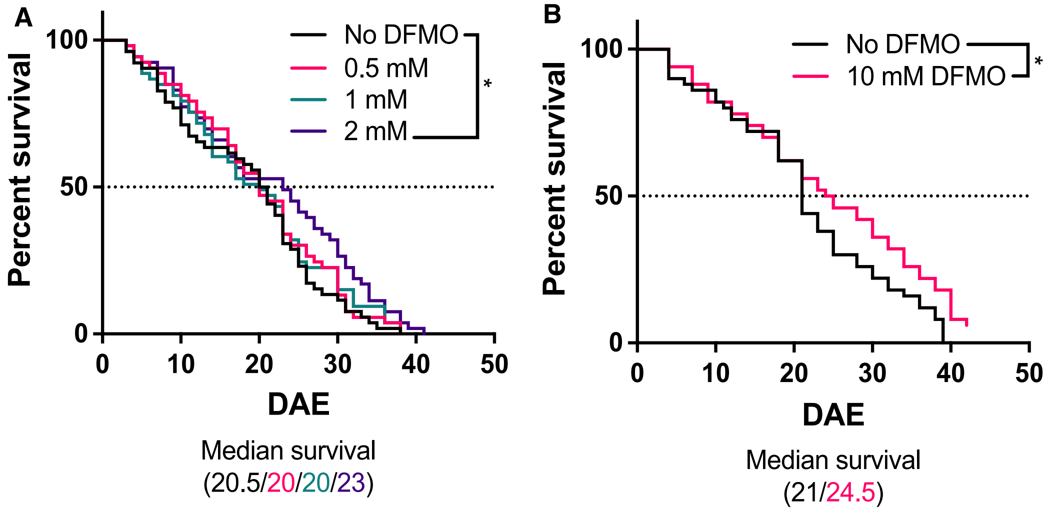

Figure EV3. DFMO extends lifespan in female *dSms*$^{-/-}$ Drosophila.

The indicated concentration of DFMO was administered in the feed and survival was observed.
A *n* = 52, 50, 53, and 53 in the 0, 0.5, 1, and 2-mM DFMO treatment groups, respectively.
B *n* = 50 and 53 in the 0 and 10-mM DFMO treatment groups, respectively.

Data information: The resulting survival curves were compared using the log-rank (Mantel–Cox) test. (WT vs. 2 mM DFMO, $P = 0.0312$; WT vs. 10 mM DFMO, $P = 0.038$). DAE, days after eclosure.

© 2023 The Authors
