# Supplementary material for: Difluoromethylornithine rebalances aberrant polyamine ratios in Snyder–Robinson syndrome
Source: EMBO Mol Med. 2023 Sep 13;15(11):e17833. doi: 10.15252/emmm.202317833 (PMC10630878; doi:10.15252/emmm.202317833)
Supplement: Supplementary file 2 — PDF+ [file EMMM-15-e17833-s003.pdf]

SOURCE DATA TRANSPARENT PROCESS OPEN ACCESS

EMBO Molecular Medicine

Article

Difluoromethylornithine rebalances aberrant polyamine ratios in Snyder–Robinson syndrome

Tracy Murray Stewart[1,*], Jackson R Foley[1], Cassandra E Holbert[1], Maxim Khomutov[2], Noushin Rastkari[1], Xianzun Tao[3], Alex R Khomutov[2], R Grace Zhai[3] & Robert A Casero, Jr[1]

Abstract

Snyder–Robinson syndrome (SRS) results from mutations in spermine synthase (SMS), which converts the polyamine spermidine into spermine. Affecting primarily males, common manifestations of SRS include intellectual disability, osteoporosis, hypotonia, and seizures. Symptom management is the only treatment. Reduced SMS activity causes spermidine accumulation while spermine levels are reduced. The resulting exaggerated spermidine:spermine ratio is a biochemical hallmark of SRS that tends to correlate with symptom severity. Our studies aim to pharmacologically manipulate polyamine metabolism to correct this imbalance as a therapeutic strategy for SRS. Here we report the repurposing of 2-difluoromethylornithine (DFMO), an FDA-approved inhibitor of polyamine biosynthesis, in rebalancing spermidine:spermine ratios in SRS patient cells. Mechanistic *in vitro* studies demonstrate that, while reducing spermidine biosynthesis, DFMO also stimulates the conversion of spermidine into spermine in hypomorphic SMS cells and induces uptake of exogenous spermine, altogether reducing the aberrant ratios. In a *Drosophila* SRS model characterized by reduced lifespan, DFMO improves longevity. As nearly all SRS patient mutations are hypomorphic, these studies form a strong foundation for translational studies with significant therapeutic potential.

Keywords alpha-methylated polyamine analogue; eflornithine; S-adenosylmethionine decarboxylase; spermidine; spermine synthase
Subject Categories Genetics, Gene Therapy & Genetic Disease; Metabolism
DOI 10.15252/emmm.202317833 | Received 10 April 2023 | Revised 18 August 2023 | Accepted 30 August 2023 | Published online 13 September 2023
EMBO Mol Med (2023) 15: e17833
See also: **SK Gilmour** (November 2023)

Introduction

Spermine synthase (SMS; EC 2.5.1.22) is the sole source of *de novo* spermine (SPM) generation in mammalian cells (Pegg, 2009). The final biosynthetic step of the mammalian polyamine pathway, SMS catalyzes the transfer of an aminopropyl group from decarboxylated S-adenosylmethionine (dcSAM) to spermidine (SPD), thereby forming SPM (Fig 1; Pegg & Michael, 2010). Germline mutations of the *SMS* gene result in Snyder–Robinson syndrome (SRS; OMIM #309583), a recessive X-linked intellectual disability syndrome predominantly affecting males (Snyder & Robinson, 1969; Arena *et al*, 1996; Cason *et al*, 2003) and associated with developmental delay, osteoporosis, muscle hypotonia, seizures, and a variety of less common symptoms (Schwartz *et al*, 2011; Starks *et al*, 2018). Medical management of SRS targets these manifestations (Schwartz *et al*, 1993), as there is no current treatment that targets the underlying biochemical cause of the disease.

Despite extracellular sources of SPM, including dietary consumption (Muñoz-Esparza *et al*, 2019), a defining characteristic of SRS-affected cells and tissues is an elevated SPD/SPM ratio that does not appear to benefit from dietary SPM supplementation at the organismal level (Mackintosh & Pegg, 2000; Cason *et al*, 2003; de Alencastro *et al*, 2008; Becerra-Solano *et al*, 2009; Wang *et al*, 2009; Schwartz *et al*, 2011; Peron *et al*, 2013; Albert *et al*, 2015; Larcher *et al*, 2019). The degree to which this ratio is altered in individual patients depends on the specific *SMS* mutation and appears to correlate with SMS enzymatic activity. Although existing data are insufficient to conclude a genotype–phenotype correlation, the pathogenic variants associated with severe disease are consistent with those causing the greatest rise in SPD/SPM ratio in our studies, including the G56S and Q148R variants as well as the single patient identified thus far with complete loss of SMS activity (M303K*fs3), who died shortly after birth (Schwartz *et al*, 1993; Larcher *et al*, 2019). A summary, including ratios, of patient cell lines used in this study can be found in Table 1. Additionally, a detailed collection of the clinical features of the individual patients was recently published by Larcher *et al* (2019).

The mammalian polyamines, including SPM, SPD, and their precursor putrescine (PUT), contribute to critical cellular functions that necessitate strict homeostatic control over their intracellular levels (Pegg, 2009). Polyamine transport is generally downregulated in the presence of high intracellular polyamine concentrations (Seiler *et al*, 1996), suggesting that SRS-affected cells might not transport extracellular SPM. However, we have shown that SPM transport activity in SRS patient-derived lymphoblastoid and

1 Sidney Kimmel Comprehensive Cancer Center, Johns Hopkins School of Medicine, Baltimore, MD, USA
2 Engelhardt Institute of Molecular Biology, Russian Academy of Sciences, Moscow, Russia
3 Department of Molecular and Cellular Pharmacology, University of Miami Miller School of Medicine, Miami, FL, USA
*Corresponding author. Tel: +1 (410) 955 4639; Fax: +1 (410) 614 9884; E-mail: tmurray2@jhmi.edu

© 2023 The Authors. Published under the terms of the CC BY 4.0 license.

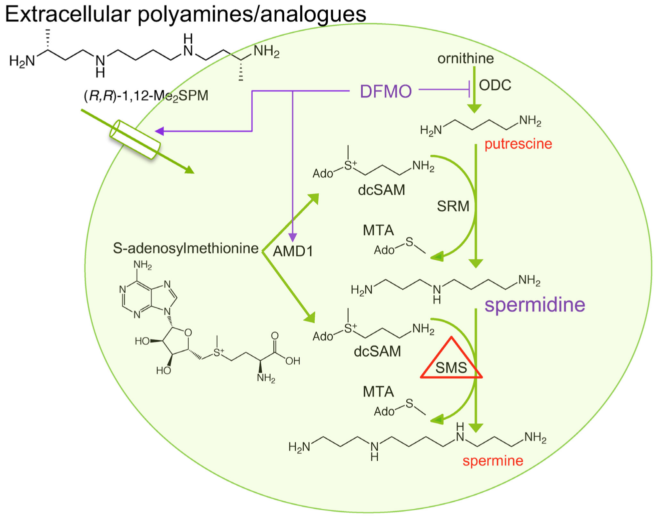

Figure 1. Effects of hypomorphic SMS and DFMO on the mammalian polyamine metabolic pathway in SRS patient-derived cells.

Putrescine is derived from ornithine via ornithine decarboxylase (ODC), the first rate-limiting step in polyamine biosynthesis. S-adenosylmethionine decarboxylase (AMD1) activity provides the aminopropyl donor dcSAM for use by spermidine synthase (SRM) and spermine synthase (SMS) to create spermidine and spermine, respectively. In SRS, hypomorphic SMS function causes spermidine accumulation while spermine levels are reduced. DFMO is an irreversible suicide inhibitor of ODC. This block of polyamine biosynthesis gradually reduces polyamine pools, triggering compensatory uptake of extracellular polyamines and induction of AMD1 activity that favors conversion into the higher polyamines (spermidine and spermine). MTA, methylthioadenosine.

fibroblast cells is as functional as that of wild-type *SMS* cells (Murray-Stewart *et al*, 2018, 2020). Importantly, in response to SPM import, SRS-affected cell lines reduce their excessive SPD pools to within normal levels, indicating that polyamine homeostatic control mechanisms also remain functional. However, the fact remains that SPM is a common dietary component that fails to rescue the SRS phenotype *in vivo*, suggesting that improvements to SPM bioavailability may enhance efficacy.

We therefore proposed the use of 2-difluoromethylornithine (DFMO) as a treatment strategy targeting the aberrant SPD/SPM ratio by reducing spermidine biosynthesis while enhancing SPM production and extracellular SPM import. DFMO is a well-studied, clinically approved, irreversible inhibitor of ornithine decarboxylase (ODC), the first rate-limiting step of polyamine biosynthesis (Fig 1; Meyskens Jr. & Gerner, 1999; Bassiri *et al*, 2015; LoGiudice *et al*, 2018). ODC activity produces the diamine PUT, which is the precursor for SPD production via SPD synthase (SRM). Importantly for our studies, cells respond to this inhibition of polyamine biosynthesis by upregulating two compensatory mechanisms: (i) increased production of the aminopropyl donor, dcSAM, to facilitate synthesis of SPM, and (ii) increased uptake of polyamines from the extracellular environment. Through these mechanisms, we reasoned that exposing SRS-affected cells to DFMO would improve the aberrantly elevated SPD/SPM ratio. We have therefore used lymphoblastoid and fibroblast cell lines from SRS patients as well as a *Drosophila* model of SRS to investigate the effects of DFMO exposure on proliferation, intracellular polyamine concentrations, extracellular polyamine uptake, polyamine metabolic enzyme activity, and phenotype reversal. Together, our results indicate that DFMO represents a potential treatment for SRS patients that targets the underlying biochemical aspects of the pathology. As DFMO is an FDA-approved drug with a history of safe administration, our results strongly support its further clinical development in the context of SRS.

Table 1. Variants and ratios of SRS patient-derived cell lines in the current study.

DNA variant	Protein	SPD/SPM ratio means ± s.e.m. ($n \geq 2$)	References
WT	WT	1.75 ± 0.18 (L)<br>0.54 ± 0.07 (f)	
c.166g>a	G56S	13.68 ± 0.78 (L)	de Alencastro *et al* (2008)
c.329+5g>a	Truncated, novel protein with some WT	7.39 ± 1.20 (L)<br>1.50 ± 0.29 (f)	Arena *et al* (1996), Cason *et al* (2003), Snyder & Robinson (1969)
c.335c>t	P112L	1.74 ± 0.08 (f)	Peng *et al* (2016)
c.443a>g	Q148R	9.43 ± 0.69 (f)	Albert *et al* (2015)
c.449t>c	I150T	2.94 ± 0.58 (f)	Zhang *et al* (2010)
c.831g>t	L277F	4.75 ± 0.83 (f)	Starks *et al* (2018)
c.906-909del	M303K*fs3	N/A[a] (f)	Larcher *et al* (2019)

(f), fibroblast; (L), lymphoblast; WT, wildtype.
[a]Ratio of N/A is due to complete lack of SPM.

Results

DFMO reduces the SPD/SPM ratio in SRS patient cells

As DFMO inhibits the first step in polyamine biosynthesis, we anticipated a reduction in intracellular SPD pools upon treatment of SRS cells. SRS patient-derived lymphoblastoid and fibroblast cell lines representing a panel of *SMS* variants were exposed to DFMO followed by HPLC-based analyses of intracellular polyamine levels. The results revealed dose-dependent reductions in SPD/SPM ratios that reached those determined in cells with wild-type *SMS* (Figs 2A, bottom panel, and EV1). As expected, decreased SPD concentrations contributed to this reduction (Fig 2A, top). However, these data also indicated increases in SPM concentrations, suggesting enhanced conversion of SPD into SPM by these defective SMS enzymes in the presence of DFMO.

In response to perturbations in polyamine biosynthesis, cells employ compensatory homeostatic control mechanisms (Casero Jr. *et al*, 2018). We considered that SRS-affected cells are likely responding to ODC inhibition by upregulating a second biosynthetic enzyme, S-adenosylmethionine decarboxylase (AMD1, Fig 1). Treatment with DFMO (96 h, 0.2 mM) strongly induced AMD1 activity in lymphoblastoid cells from SRS patients (Fig 2B), suggesting increased availability of its reaction product, dcSAM, which is the aminopropyl group donor for SPM synthesis through the hypomorphic SMS enzymes. LC/MS analysis of intracellular dcSAM

 © 2023 The Authors

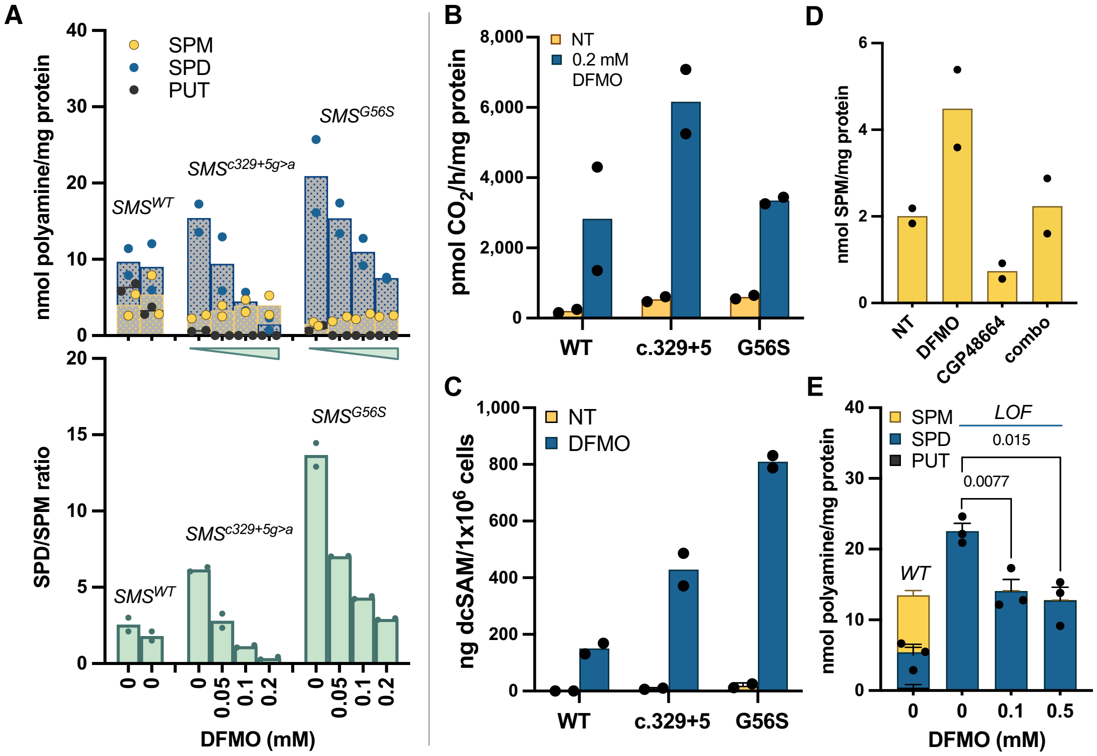

Figure 2. Mechanism of action of DFMO in SRS patient-derived lymphoblastoid cells.

A DFMO treatment reduced intracellular SPD levels and increased SPM (top), correcting the SPD/SPM ratio to *SMS*^{WT} levels (bottom). SRS cells were treated with increasing concentrations of DFMO as indicated below the bottom panel. Untreated cell lines from two different *SMS*^{WT} healthy donors are included for comparison. Individual polyamine concentrations from two independent biological replicates (each performed in technical duplicate) are plotted with means indicated by column height; concentrations are presented as nmol polyamine per mg protein in the lysate in the top panel, with SPD/SPM ratio calculations plotted in the bottom panel.

B DFMO treatment (96 h, 0.2 mM) induced AMD1 enzymatic activity, presented as pmol of CO_2 released in 1 h per mg of protein in the cell lysate. Column heights indicate the means of two biological replicates (each individually indicated as the mean of triplicate measurements). NT, no treatment.

C DFMO treatment (96 h, 0.2 mM) increased intracellular concentrations of dcSAM, the reaction product of AMD1 essential for SPM biosynthesis. LC–MS/MS was used to quantify dcSAM levels in DFMO-treated lymphoblasts. Columns indicate the means of independent biological experiments denoted as symbols.

D Cotreatment with an AMD1 inhibitor (1 μM CGP48664, 96 h) prevented the DFMO-mediated production of SPM in *SMS*^{G56S} cells (columns indicate the means of individually plotted biological replicates, each of which represents the average of duplicate measurements).

E SRS patient cells with complete loss of SMS function (“LOF”, *SMS*^{M303K*fs3}) responded to DFMO treatment with significantly reduced SPD production but did not produce SPM, supporting the hypomorphic versions of SMS as the source of DFMO-mediated SPM production. (n = 3, duplicate measurements, ordinary one-way ANOVA, Dunnett’s correction for multiple comparisons). WT, wild-type *SMS*.

Data information: Columns and bars indicate the means with s.e.m.
Source data are available online for this figure.

levels further support this hypothesis: dcSAM levels were elevated with DFMO treatment in all cell lines examined, with substantially greater levels evident in the SRS lines compared to WT (Fig 2C). In the absence of DFMO, dcSAM was below the limit of detection in cells with WT *SMS*, while SRS patient lymphoblasts contained readily measurable amounts (Fig EV2A), consistent with a previous report (Pegg *et al*, 2011). Levels of unmodified SAM did not differ among the untreated cell lines. However, treatment of the *SMS*^{G56S} SRS line with DFMO was associated with a reduction of SAM concentrations (Fig EV2B). Further, cotreatment with the AMD1 inhibitor CGP48664 (Regenass *et al*, 1994) completely blocked the DFMO-mediated increase in SPM production in SRS cells (Fig 2D), supporting a critical role for AMD1 induction in the response to DFMO. To further confirm our hypothesis that DFMO facilitates the conversion of SPD to SPM in SRS cells containing hypomorphic variants of the SMS protein, we exposed SRS cells containing a complete loss-of-function (LOF) *SMS* variant to DFMO (Fig 2E). Consistent with DFMO-mediated inhibition of biosynthesis, SPD was reduced; however, no SPM was evident in these cells regardless of DFMO dosage. Together, these data indicate a mechanism of action for DFMO in cells with hypomorphic SMS that includes reduced levels of SPD in conjunction with AMD1-mediated increased availability of dcSAM that facilitates SPM biosynthesis by the defective SMS enzyme (Fig 1).

SMS variants alter sensitivity to DFMO-mediated growth inhibition

DFMO can be anti-proliferative, particularly in rapidly dividing cells with increased dependence on polyamines. SRS or healthy donor fibroblast cell lines were incubated for 96 h in the presence of increasing concentrations of DFMO (Fig 3A). Cell lines chosen for

© 2023 The Authors

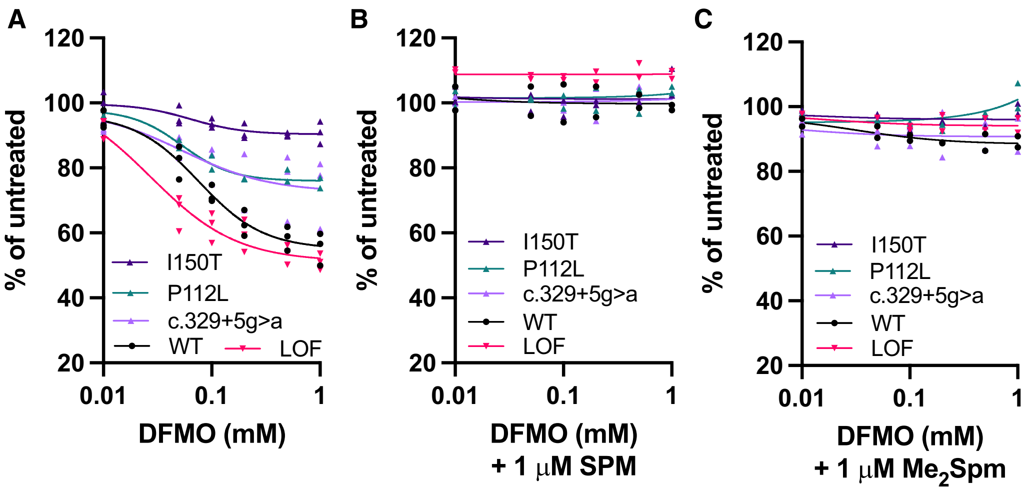

Figure 3. Effects of DFMO on SRS patient fibroblasts: mutation-specific sensitivity and rescue by spermine.

Dose responses of fibroblast cell lines to DFMO were plotted by non-linear regression for comparison of sensitivity and IC_{50} values.

A Cells from healthy donors (WT) or SRS patients (indicated by *SMS* variant) were grown for 96 h in the presence of increasing doses of DFMO. Biological $n = 3$, each in technical triplicate with mean values plotted. Comparison of fits using extra sum-of-squares F test indicate the curves of each *SMS* variant treated with DFMO alone significantly differed from the WT curve ($P < 0.0001$).

B Growth of cells from healthy donors (WT) or SRS patients exposed to DFMO was rescued by combination with 1 µM SPM.

C Growth of cells from healthy donors (WT) or SRS patients exposed to increasing doses of DFMO was rescued by 1 µM of the spermine mimetic 1,12-Me_2SPM.

Data information: All treatments included 1 mM aminoguanidine to inhibit extracellular polyamine oxidation. Cell viability was measured by fluorescence following incubation with CellTiter Blue. Results are presented as percentages relative to untreated cells, and data points represent the means of independent biological experiments, each performed in triplicate wells. No differences existed between curves in the presence of SPM or mimetic. LOF, loss-of-function.

Source data are available online for this figure.

these studies represented severely affected (I150T) and more mildly affected (c.329+5g>a, P112L) patients with varying elevations in ratios resulting from the individual *SMS* mutations (Table 1; Murray Stewart *et al*, 2020). Despite similar growth rates, these hypomorphic SRS cell lines were found to be significantly more resistant to the growth inhibitory effects of DFMO than WT cells from healthy donors (shown are combined data from two different WT cell lines). In contrast, cells with a complete LOF mutation (M303K*fs3) in *SMS* demonstrated increased sensitivity to DFMO compared to WT cells. Specifically, the hypomorphic cell lines differed in the maximum inhibition of growth caused by DFMO exposure, ranging from approximately 10 to 27%, while WT cells demonstrated a maximum of approximately 46%. Although the maximum growth inhibition of the LOF mutant was similar to that of WT (~50%), a left-shifted curve was observed that was reflected in a reduction of the IC_{50} value from 79 µM in SMS^{WT} cells to 27 µM in the LOF mutant line (Table 2). Regardless of the degree of growth inhibition resulting from DFMO exposure, proliferation was completely rescued when low doses of SPM or its mimetic (1,12-Me_2SPM) were included during treatment (Fig 3B and C).

Table 2. Differential sensitivity of SMS variant fibroblast cell lines to DFMO (t = 96 h).

SMS genotype	WT	I150T	P112L	c.329+5	LOF
IC_{50} (µM)	76.3	63.7	53.2	54.2	27.3
% max inhibition	45.0	9.7	24.0	27.5	48.8

DFMO induces uptake of and cooperates with extracellular spermine or a mimetic to reduce the SPD/SPM ratio

We previously established that both lymphoblastoid and fibroblast cell lines originating from SRS patients could import SPM from the extracellular environment to restore a more normal polyamine distribution profile (Murray-Stewart *et al*, 2018, 2020). Inhibition of polyamine biosynthesis with inhibitors such as DFMO is known to evoke a compensatory increase in polyamine import activity. We therefore investigated the effects of treating SRS cells with DFMO in the presence of physiologically relevant concentrations of extracellular SPM. Aminoguanidine (1 mM) was included in the culture medium to inhibit the extracellular oxidation of SPM (Holbert *et al*, 2020). In SRS lymphoblasts with the SMS^{G56S} mutation, providing exogenous SPM in a concentration range relevant to that reported in human plasma (Liu *et al*, 2012) did not reduce the concentration of SPD or total polyamines, but it did reduce the SPD/SPM ratio (Fig 4A). However, in the presence of DFMO, these same SPM concentrations more effectively restored intracellular SPM concentrations, decreased SPD levels, and returned the SPD/SPM ratio to that observed in SRS WT cells.

To clarify the effect of DFMO treatment on polyamine uptake, we replaced SPM with 1,12-Me_2SPM, a SPM mimetic that is resistant to amine oxidase activity and is capable of supporting cellular proliferation in the presence of DFMO (Lakanen *et al*, 1992; Byers *et al*, 1994; Jarvinen *et al*, 2005). Me_2SPM is also easily distinguished from endogenous SPM by HPLC, thus allowing precise measurement of intracellular concentrations. The presence of DFMO

© 2023 The Authors

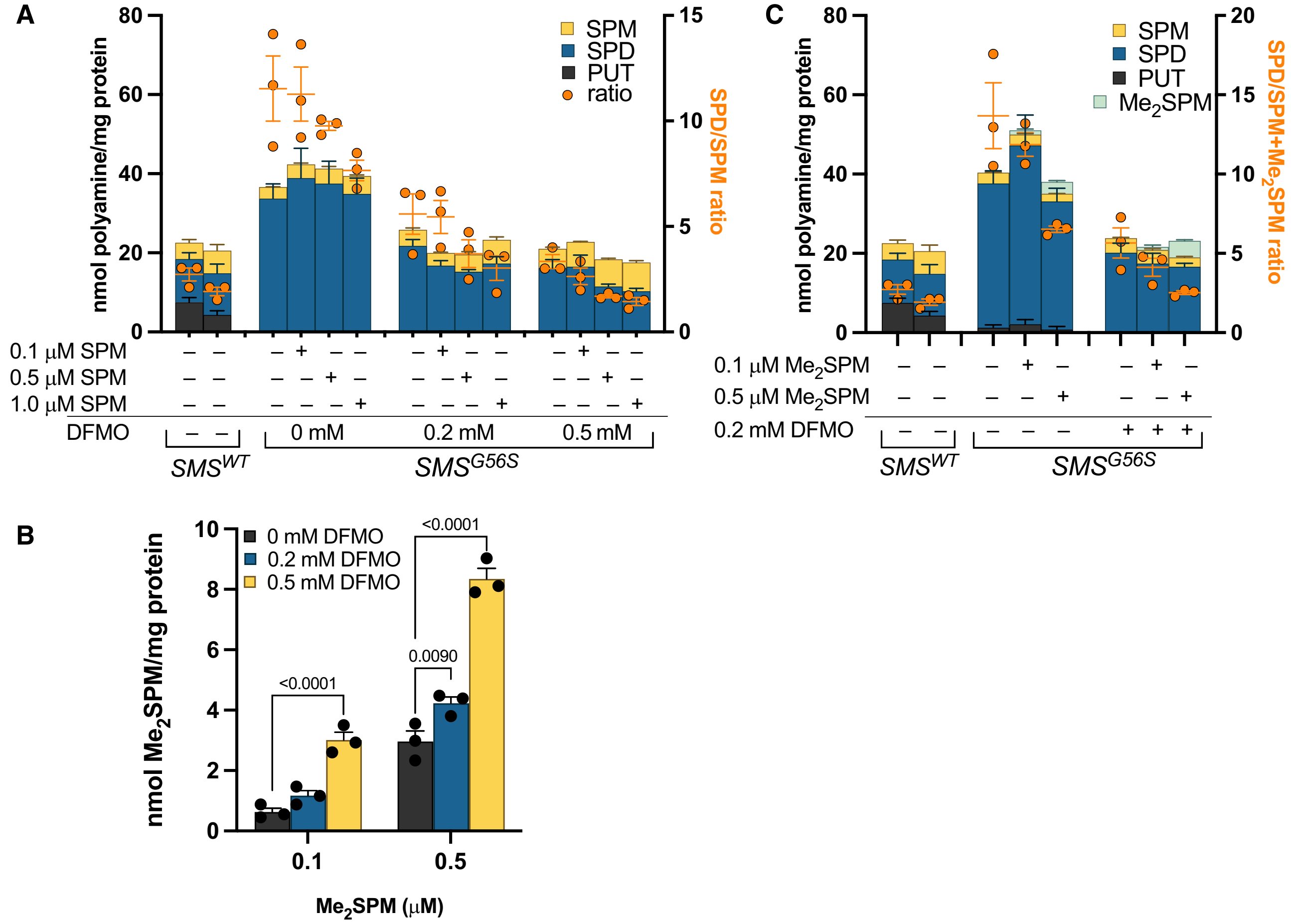

Figure 4. SPD/SPM ratio reduction by DFMO is enhanced in the presence of extracellular spermine or a spermine mimetic.

SMS^{G56S} lymphoblasts were exposed to physiologically relevant doses of extracellular SPM or the SPM mimetic Me_2SPM in the presence of DFMO (with 1 mM aminoguanidine to inhibit bovine serum amine oxidases) for 96 h, followed by polyamine pool determinations by HPLC. Untreated lymphoblast lines from two different *SMS*^{WT} healthy donors are included for comparison of polyamine levels and SPD/SPM ratio.

A The provision of exogenous SPM improved the response to DFMO by reducing SPD and increasing SPM, together reducing the SPD/SPM ratio (orange dots plotted on the right axis) beyond that with DFMO or SPM alone; $n = 3$; orange bars and error bars indicate the mean ratios with s.e.m.

B DFMO increased accumulation of Me_2SPM. Increases in intracellular concentrations of Me_2SPM were measured by HPLC following cotreatment with DFMO ($n = 3$, ordinary two-way ANOVA with Dunnett's multiple comparisons test).

C Low-dose DFMO improved response to Me_2SPM by reducing SPD, improving uptake, and returning the ratio (orange dots plotted on the right axis) to that of *SMS*^{WT} cells ($n = 3$; orange bar and error bars indicate the mean ratios with s.e.m.).

Data information: In all panels, intracellular polyamine levels are plotted as columns on the left axis and indicate the means ± s.e.m. of "*n*" biological experiments, each performed in duplicate.
Source data are available online for this figure.

increased the accumulation of intracellular Me_2SPM by approximately three-fold (Fig 4B), thereby reducing the dose required and potentially reducing off-target effects. Polyamine pool analyses confirmed that DFMO and Me_2SPM cooperatively reduced the elevated levels of SPD, total polyamines, and the SPD/SPM ratio in SRS cells (Fig 4C), indicating the combination of DFMO and Me_2SPM as a potential treatment option.

DFMO extends lifespan in a *Drosophila* model of SRS

We used a previously reported *Drosophila* model of SRS to determine the potential for phenotype reversal by DFMO *in vivo* (Li *et al*, 2017). The phenotype of this model includes a significantly shortened lifespan that can be reversed by overexpression of wild-type human *SMS*. As the *dSMS* gene is autosomal in flies, both male and female flies were included in the study. The addition of increasing concentrations of DFMO to the feed of *dSms*$^{-/-}$ flies increased lifespan in a dose-dependent manner (Figs 5A and EV3). Survival of male flies was significantly extended starting at 1 mM DFMO, with the highest dose, 10 mM, lengthening median life span from 21 to 35 days (Fig 5B). Survival in female flies was also significantly extended, albeit to a lesser extent (Fig EV3). These results indicate the feasibility of oral administration of DFMO in eliciting systemic, phenotype-reversing effects at the organismal level, and together

© 2023 The Authors

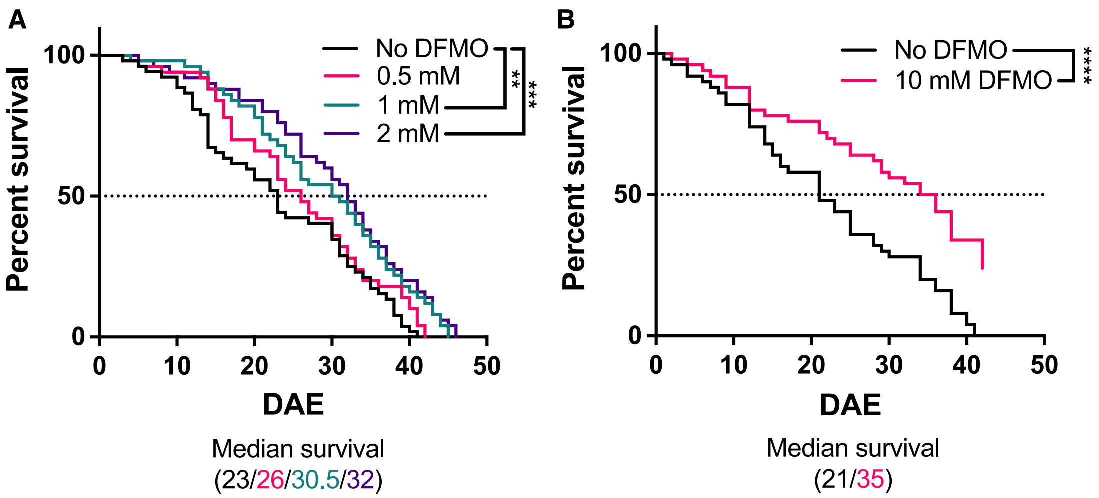

Figure 5. DFMO extends lifespan in male *dSms*$^{-/-}$ *Drosophila*.

The indicated concentration of DFMO was administered in the feed and survival was observed.

A *n* = 52 in the control group, and 50 in each of the 0.5, 1, and 2-mM DFMO treatment groups.

B *n* = 50 each in the 0 and 10-mM DFMO treatment groups.

Data information: The resulting survival curves were compared using the log-rank (Mantel–Cox) test; $^{**}P = 0.0041$, $^{***}P = 0.0008$, $^{****}P < 0.0001$. DAE, days after eclosure. Source data are available online for this figure.

with our biochemical data in patient cell lines, support the use of DFMO clinically in the context of SRS.

Discussion

Our studies indicate the potential repurposing of DFMO as a treatment strategy for males with SRS. To date, only a single patient has been identified with complete loss of SMS activity (Larcher *et al*, 2019). Partial LOF, hypomorphic variants are much more common, which often interfere with the dimerization required for efficient aminopropyltransferase activity (Wu *et al*, 2008; Zhang *et al*, 2010; Peng *et al*, 2016). Our treatment of SRS cells in culture with DFMO demonstrates that the hypomorphic SMS enzymes can indeed convert SPD into SPM, a reaction that depends upon induction of AMD1 (Fig 2A–D). In contrast, treatment of the SMS LOF line reduces the SPD concentration but fails to produce SPM (Fig 2E). Increased availability of dcSAM to the hypomorphic enzyme likely contributes to this reaction; however, whether the SMS enzyme itself is affected by the DFMO treatment remains to be determined. It should be noted that although DFMO is a specific and irreversible inhibitor of ODC, the synthesis and rapid turnover of ODC results in some PUT generation which, in the presence of high dcSAM is rapidly converted to SPD and ultimately SPM, if functional SMS is present (Glass & Gerner, 1986).

Decarboxylated SAM, resulting from AMD1 activity, provides the aminopropyl donor for transfer onto PUT or SPD acceptor molecules, thereby forming SPD or SPM, respectively (Fig 1). The increase in basal AMD1 enzyme activity observed in the SRS lymphoblasts compared to those with wild-type SMS is consistent with the increased generation of dcSAM. Of the polyamines, SPM most potently downregulates AMD1 activity (Pegg, 1984; Shantz *et al*, 1992), suggesting that the reduced SPM levels characteristic of SRS cells may contribute to these results. The reduced use of dcSAM by the variant SMS enzymes further contributes to its accumulation. Similar results have been reported in cells from SRS patient families including those with the *SMS*^{G56S} variant and the *SMS*^{I150T} variant, not included in the current study (Pegg *et al*, 2011). In Gy mice, which completely lack the *Sms* gene, significantly elevated dcSAM levels were detected in the brain, kidney, liver, and heart (Pegg *et al*, 2011).

Though compensatory responses to polyamine biosynthesis inhibition by DFMO has been well documented in a variety of cell types (Mamont *et al*, 1982; Pegg, 1984; Shantz *et al*, 1992), it has not been previously investigated in SRS patient cells. DFMO treatment greatly induced AMD1 activity in lymphoblastoid cell lines. While the extent of induction was similar between WT cells and the G56S variant, induction in the c.329+5 variant significantly exceeded either of these (Fig 2B). However, the resulting accumulation of the AMD1 product, dcSAM, did not correlate with AMD1 activity, but rather inversely correlated with SMS activity, as indicated by the resulting positive correlation with SPD/SPM ratio (Fig 2A, bottom panel and C; note that SPD is entirely depleted by DFMO in WT cells, thus no ratio is indicated). By inhibiting ODC, DFMO reduces the production of PUT and SPD, thereby reducing the pool of potential acceptors of dcSAM in aminopropyltransferase reactions. This substrate limitation, combined with active SPD and SPM synthesis, accounts for the lower dcSAM accumulation in WT cells. Though the elevated SPD levels in SRS cells reduces the substrate limitation, the lack of efficient aminopropyltransferase activity by the SMS variants allows additional dcSAM buildup that is available for transfer mainly onto SPD by SMS. The c.329+5g>a *SMS* variant disrupts a splice site, resulting in a truncated protein in addition to the full-length, functional SMS protein due to read-through (Cason *et al*, 2003). Consequently, cells with this variant have measurable SMS activity that

 © 2023 The Authors

maintains a SPD/SPM ratio 3–4 times that of WT controls (Fig 2A, bottom panel and Cason *et al*, 2003). DFMO facilitates the conversion of SPD into SPM at concentrations as low as 50 μM in both lymphoblasts and fibroblasts with this variant (Figs 2A and EV1). This increased utilization of dcSAM in the SMS reaction is likely responsible for the difference in dcSAM measured between the 2 SRS cell lines (Figs 2C and EV2A). The G56S variant of SMS possesses very little enzymatic activity, resulting in a ratio approximately 8-fold greater than controls (Table 1, Fig 2A, bottom and de Alencastro *et al*, 2008) and requiring higher DFMO concentrations to appropriately reduce the ratio, although it should be noted that the 0.2 mM concentration of DFMO used remains much lower than the doses commonly used to inhibit biosynthesis in cultured cells. The evident inverse association between intracellular SPM and dcSAM concentrations following DFMO treatment may also reflect negative regulation of AMD1 activity by SPM *in situ* (Shantz *et al*, 1992). These results also suggest that clinical use of DFMO to treat SRS patients may need to be tailored to the individual depending on the amount of residual SMS activity or SPD/SPM ratio. Indeed, in cell lines with the c.329+5g>a variant (Figs 2A and EV1), exposure to DFMO ultimately depleted SPD pools, which may also be detrimental. However, we anticipate the *in vivo* situation to be less responsive in this regard due to compensatory uptake of extracellular PUT and/or SPD. Future studies in relevant mammalian animal models should clarify this potential. Regardless, the results of these studies indicate that a functional readout of SMS activity, such as SPD/SPM ratio, should be an important consideration in DFMO clinical trial design.

Though dcSAM levels are elevated in SRS patient cells, no evident differences were detected in baseline SAM levels (Fig EV2B), as previously observed (Pegg *et al*, 2011). However, following treatment with DFMO, a clear decrease in SAM was noted in the *SMS*^{G56S} cell line, concurrent with this cell line demonstrating the greatest accumulation of dcSAM. This result warrants further consideration and potentially indicates a need for SAM supplementation in the clinical context, as SAM is an important methyl group donor for many reactions.

Our studies indicate the potential for DFMO to improve the low bioavailability of SPM that is apparent with its oral ingestion in SMS-deficient animals (Mackintosh & Pegg, 2000; Wang *et al*, 2009). Additionally, while DFMO cannot stimulate the production of SPM by SMS LOF variants, it does stimulate uptake of SPM, as indicated by our rescue experiments (Fig 3), thereby providing a source of SPM to lower the SPD/SPM ratio. These rescue experiments also indicated that the most common SRS-associated mutations, all of which are hypomorphic, increase resistance to the growth inhibitory effects of DFMO. Although, as extracellular sources of SPM include the diet and microbiota (Muñoz-Esparza *et al*, 2019; Holbert *et al*, 2022), growth inhibition by DFMO is not anticipated to be a treatment limitation in SRS. However, an early mouse model that completely lacked the chromosomal region including the *Sms* gene was extremely sensitive to DFMO, which had detrimental effects (Wang *et al*, 2009). Whether this resulted from the lack of Sms activity or an alternative function is unclear, but caution is warranted when considering patient inclusion criteria for clinical trial design. Conversely, the *Drosophila* strain used in these studies also had complete lack of Sms function. These flies were viable and responded positively to the addition of DFMO. Notably, the standard media used for *Drosophila* studies includes measurable SPM (approximately 4 nmol/mg), suggesting its enhanced uptake by the *dSms*$^{-/-}$ flies may be responsible for the extended lifespan in response to DFMO. DFMO also improved the uptake and response to *R*,*R*-1,12-Me_2SPM (Fig 4B and C), a metabolically stable SPM mimetic we previously reported to improve the SPD/SPM ratio in SRS patient cell lines (Murray Stewart *et al*, 2020). Alpha-methylation of SPM prevents its acetylation and/or oxidation via the polyamine catabolic enzymes, cellular monoamine and diamine oxidases, as well as serum amine oxidases (Lakanen *et al*, 1992; Hyvonen *et al*, 2007). The production of toxic byproducts via these reactions limits the use of native SPM as a treatment *in vivo*, especially when administered to animals by intravenous or intraperitoneal routes (Tabor & Rosenthal, 1956; Til *et al*, 1997; Pegg, 2013). Thus, as Me_2SPM is amenable to more direct modes of delivery, it could serve as an alternative to replace SPM. Although Me_2SPM effectively reduced SPD/SPM ratios when administered to intraperitoneally mice, evidence of toxicity was observed at higher concentrations (Murray Stewart *et al*, 2020). Our data indicating a combined effect of DFMO and Me_2SPM importantly imply that, when used together, lower doses of each will be required to elicit the desired effect, thereby reducing the potential for adverse effects. A mouse model of SRS, which harbors the clinically relevant *Sms*^{G56S} mutation, recently became available (The Jackson Laboratory, strain #033707). Our plans for future studies include determining the effects of DFMO, SPM, and Me_2SPM on these mice; however, a patient-relevant phenotype must first be identified.

DFMO is clinically approved for the treatment of African trypanosomiasis (Bacchi, 2009), and its use as a maintenance therapy for pediatric neuroblastoma patients has provided valuable data on safety and long-term administration (Saulnier Sholler *et al*, 2015; Lewis *et al*, 2020). DFMO has a decades-long history of safe administration in clinical trials and has recently been repurposed to treat patients with Bachmann–Bupp syndrome (BABS), a newly identified genetic disorder resulting from an ODC gain-of-function variant (Bupp *et al*, 2018; Rajasekaran *et al*, 2021). The presentation of BABS emulates that of SRS in several aspects, including developmental delay, hypotonia, dysmorphic features, and non-specific brain MRI findings (VanSickle *et al*, 2021), most of which dramatically improved with DFMO treatment (Rajasekaran *et al*, 2021). Notably, both BABS and neuroblastoma patients receive multiple oral doses of DFMO daily on a continuing basis, representing patient populations and dosing schedules similar to those anticipated in an SRS clinical trial. Our studies in SRS patient-derived cell lines indicate that DFMO effectively reverses the aberrantly elevated SPD/SPM ratio that is the main biochemical hallmark of SRS. Additionally, adding DFMO to the feed of *Drosophila* lacking *dSms* significantly extends lifespan, a main phenotype in this SRS model (Li *et al*, 2017). Taken together, our results indicate that repurposing DFMO for the treatment of SRS is a valid therapeutic strategy that targets the underlying biochemistry driving the SRS phenotype, thereby supporting further clinical translation of DFMO into this population.

Materials and Methods

Cell lines, culture conditions, and compounds

Lymphoblastoid and dermal fibroblast cell lines were derived from male SRS patients or healthy donors at the Greenwood Genetic

Center (Greenwood, SC) and were previously described (Cason et al, 2003; de Alencastro et al, 2008; Becerra-Solano et al, 2009; Murray-Stewart et al, 2018). Our cell line panels include seven unique *SMS* variants (Table 1), representing 35% of the total published variants (Schwartz et al, 1993), along with sex-matched cell lines expressing wild-type *SMS*. Lymphoblastoid lines were maintained in RPMI 1640 containing 15% fetal bovine serum (Gemini Bio-Products, Sacramento, CA), 2 mM glutamine, sodium pyruvate, non-essential amino acids (NEAA), and penicillin/streptomycin at 5% CO_2 and 37°C. Fibroblast cell lines were maintained in DMEM containing 15% fetal bovine serum, L-glutamine, sodium pyruvate, NEAA, and antibiotics at 5% CO_2 and 37°C. All cell lines were determined to be free of mycoplasma within 6 months of experimentation (MycoAlert, Lonza, Walkersville, MD). (*R*,*R*)-1,12-Me_2SPM was synthesized previously (Grigorenko et al, 2007) and DFMO was provided by Professor Patrick M. Woster (Medical University of South Carolina).

Polyamine concentration determinations and enzyme activity assays

Intracellular polyamine concentrations of cell or tissue lysates were determined by HPLC following acid extraction and dansylation of the supernatant, as previously described (Kabra et al, 1986). HPLC standards included diaminoheptane (internal standard), PUT, SPD, SPM, and acetylated SPD and SPM, all of which were purchased from Sigma Chemical Co. Enzyme activity assays were performed for SAMDC using radiolabeled SAM, as previously described (Pegg, 1984). Enzyme activities and intracellular polyamine determinations are presented relative to total cellular protein, as determined using the Bio-Rad Protein Assay (Hercules, CA) with interpolation on a bovine serum albumin standard curve.

SAM and dcSAM quantification

SAM and dcSAM levels were quantified using a method developed by the Analytical Pharmacology Shared Resource of the Sidney Kimmel Comprehensive Cancer Center at Johns Hopkins. Decarboxylated SAM-d3 was purchased from Toronto Research Chemicals (North York, Ontario, CA). Unlabeled dcSAM and SAM were purchased from Sigma Chemical Co. Deionized water was obtained from a Millipore Milli-Q-UF filtration system (Milford, MA). A standard solution containing the target analytes, SAM, and dcSAM (1 mg/ml), was prepared in acetonitrile:water (50:50, v/v). These stock standard solutions were diluted with methanol:water (50:50, v/v) to prepare a mixed working solution with different concentrations. dcSAM-d3 was used as internal standard (I.S.) and prepared in water at a concentration of 15 ng/ml. Stock and working solutions were stored at 4°C for daily use.

Methanol:water (50:50, v/v) was selected as the extraction solvent and yielded good extraction recoveries for SAM and dc-SAM. First, 500 µl of the extraction solvent were added to the frozen cell pellets, the identities of which were concealed until analysis was complete. The samples were slowly thawed at room temperature. Subsequently, the cells were shock-frozen in −80°C for 10 min and thawed for a second time at room temperature. The samples were vortexed and sonicated for 60 s, then vortexed and centrifuged at 12,000 *g* for 5 min at 4°C. After centrifugation, 50 µl of each supernatant was transferred into an autosampler vial along with 20 µl of the internal standard for LCMS/MS analysis of dc-SAM. Another 50 µl of the supernatant was added to 450 µl of cold methanol, and then 50 µl of the diluted solution along with 20 µl of the internal standard was transferred into autosampler vials for LCMS/MS analysis of SAM.

Separation was achieved with a Waters Acquity UPLC BEH Amide 2.1 × 100 mm, 1.7 µm (Waters Corporation, Milford, MA). Mobile phase A was 0.1% formic acid in 10 mM of ammonium formate, and mobile phase B was 0.1% formic acid in acetonitrile. A gradient was used and mobile phase B was held at 15% with a flow rate of 0.15 ml/min for 0.5 min. It was then increased to 80% over 1.5 min and held there for 2 min, then returned to 15% B and was held there for 1 min. The column effluent was monitored using an SCIEX 6500 triple quadrupole mass-spectrometric detector (Sciex, Foster City, CA, USA) using electrospray ionization operating in positive mode. The mass spectrometer was programmed to monitor the following MRM transitions: 399.04 → 250.10 for SAM, 356.96 → 250.20 for dcSAM, and 357.97 → 250.00 for the internal standard, dcSAM-d3. Calibration curves for SAM and dcSAM were computed using the area ratio peak of the analysis to the internal standard by using a quadratic equation with a $1/x^2$ weighting function and calibration ranges of 5 to 1,000 ng/ml. For SAM, the additional 1:10 dilution was factored into the final concentration calculation.

Cell proliferation assays

Fibroblast cells were seeded at 1,000 cells/well in 96-well plates and allowed to attach overnight. Growth medium was replaced with that containing increasing concentrations of DFMO with or without 1 µM *R*,*R*-1,12-Me_2SPM or SPM in the presence of 1 mM aminoguanidine (100 µl total per well), and cells were incubated 96 h. CellTiter-Blue reagent (Promega, Madison, WI) was added (20 µl/well) followed by an additional 3-h incubation at 37°C. Fluorescence was measured in white, opaque-bottom plates at $560_{Ex}/590_{Em}$ on a SpectraMax M5 platereader (Molecular Dynamics, Sunnyvale, CA), using wells containing medium alone (no cells) as background controls. Non-linear, least squares regression curves were plotted for determination of IC_{50} values and maximal inhibition.

Drosophila studies

The *dSms* mutant fly (d*Sms*$^{-/-}$) strain has been previously characterized (Li et al, 2017; Tao et al, 2022). Flies were maintained in vials with cornmeal–molasses–yeast medium at 22°C, 65% humidity, and 12-h light/12-h dark. To measure the life span, newly eclosed flies were collected, and 10–20 randomly selected flies of the same sex were kept in vials with food with or without the indicated concentration of DFMO. Flies were transferred to new vials every week, and the number of dead flies was counted every other day.

Statistical analyses

All statistically significant differences were determined using GraphPad Prism software (v. 9.5.1, La Jolla, CA). Datasets passed Shapiro–Wilk normality tests prior to further analyses. Comparisons between untreated and treated WT and SRS cell lines were performed

© 2023 The Authors

The paper explained

Problem
Snyder–Robinson syndrome (SRS) is a debilitating genetic syndrome arising from a deficiency in the spermine synthase enzyme, which is biochemically identified as an elevation of the ratio of two polyamines—the precursor and product of this enzyme (spermidine/spermine). With no cure, treatment options aim only to manage symptoms. Our study investigated the use of pharmacological mediators of the polyamine metabolic pathway to rebalance this skewed ratio as a potential treatment for SRS.

Results
Cell lines derived from SRS patients were treated with an inhibitor of polyamine biosynthesis (difluoromethylornithine, DFMO) alone or in combination with natural spermine or a spermine mimetic. DFMO treatment reduced the aberrant ratio to within normal levels, and this effect was amplified in the presence of spermine (a common dietary component) or its mimetic. We further define the mechanism of action of DFMO in SRS cells and characterize the potential for differential responses based on specific clinical variants in the enzyme.

Impact
Our study suggests the repurposing of DFMO as an SRS treatment strategy targeting the molecular changes underlying the phenotype, providing a strong basis for translation into clinical trials, and addressing a critical unmet need in this population.

as indicated in figure legends, with correction for multiple comparisons. P-values ≤ 0.05 were considered statistically significant.

Data availability

This study includes no data deposited in external repositories.

Expanded View for this article is available online.

Acknowledgements
Our studies were made possible first and foremost by the Snyder–Robinson Foundation, the SRS patients, and their families, who provided invaluable resources and inspiration. These studies were supported by funding from the Snyder–Robinson Foundation via the Million Dollar Bike Ride of the Orphan Disease Center at the University of Pennsylvania (MDBR-20-135-SRS and MDBR-21-106-SRS to RAC and TMS) and the Chan-Zuckerberg Initiative. Funding was also provided by the National Institutes of Health National Cancer Institute (R01CA204345, R01CA235963 to RAC), the Eunice Kennedy Shriver National Institute of Child Health and Human Development (R01HD110500 to RAC and TMS), the National Institute of Neurological Disorders and Stroke (R01NS109640 to RGZ), and the Commonwealth Foundation for Cancer Research (to RAC). Synthesis of 1,12-Me_2SPM was supported by the Russian Science Foundation (Grant 17-74-20049 to ARK). Additional support was provided by the Analytical Pharmacology Shared Resource of the Sidney Kimmel Comprehensive Cancer Center at Johns Hopkins directed by Dr. Michelle Rudek [NIH grants P30CA006973 and UL1TR003098, and the Shared Instrument Grant S10RR026824] and grant number UL1TR003098 from the National Center for Advancing Translational Sciences (NCATS), a component of the National Institutes of Health (NIH), and the NIH Roadmap for Medical Research. Its contents are solely the responsibility of the authors and do not necessarily represent the official view of the NCATS or NIH. The graphical abstract was produced using bioRender.

Author contributions
Tracy Murray Stewart: Conceptualization; resources; data curation; formal analysis; supervision; funding acquisition; validation; investigation; visualization; methodology; writing – original draft; project administration; writing – review and editing. **Jackson R Foley:** Data curation; formal analysis; validation; writing – review and editing. **Cassandra E Holbert:** Formal analysis; writing – review and editing. **Maxim Khomutov:** Resources; writing – review and editing. **Noushin Rastkari:** Investigation; methodology; writing – original draft; writing – review and editing. **Xianzun Tao:** Data curation; formal analysis; validation; investigation; visualization; methodology; writing – original draft; writing – review and editing. **Alex R Khomutov:** Resources; supervision; writing – review and editing. **R Grace Zhai:** Resources; supervision; funding acquisition; methodology; writing – original draft; writing – review and editing. **Robert A Casero Jr:** Conceptualization; resources; funding acquisition; methodology; project administration; writing – review and editing.

Disclosure and competing interests statement
The Casero and Stewart Laboratory receive funding from Panbela Therapeutics, Inc. The funders had no role in the design of the study; in the collection, analyses, or interpretation of data; in the writing of the manuscript, or in the decision to publish the results.

For more information
i https://www.omim.org/entry/309583
ii https://www.snyder-robinson.org/
iii https://www.spectrumhealth.org/research-and-clinical-trials/icpd

References

Albert JS, Bhattacharyya N, Wolfe LA, Bone WP, Maduro V, Accardi J, Adams DR, Schwartz CE, Norris J, Wood T *et al* (2015) Impaired osteoblast and osteoclast function characterize the osteoporosis of Snyder–Robinson syndrome. *Orphanet J Rare Dis* 10: 27

Arena JF, Schwartz C, Ouzts L, Stevenson R, Miller M, Garza J, Nance M, Lubs H (1996) X-linked mental retardation with thin habitus, osteoporosis, and kyphoscoliosis: linkage to Xp21.3-p22.12. *Am J Med Genet* 64: 50 – 58

Bacchi CJ (2009) Chemotherapy of human African trypanosomiasis. *Interdiscip Perspect Infect Dis* 2009: 195040

Bassiri H, Benavides A, Haber M, Gilmour SK, Norris MD, Hogarty MD (2015) Translational development of difluoromethylornithine (DFMO) for the treatment of neuroblastoma. *Transl Pediatr* 4: 226 – 238

Becerra-Solano LE, Butler J, Castaneda-Cisneros G, McCloskey DE, Wang X, Pegg AE, Schwartz CE, Sanchez-Corona J, Garcia-Ortiz JE (2009) A missense mutation, p.V132G, in the X-linked spermine synthase gene (SMS) causes Snyder–Robinson syndrome. *Am J Med Genet A* 149A: 328 – 335

Bupp CP, Schultz CR, Uhl KL, Rajasekaran S, Bachmann AS (2018) Novel de novo pathogenic variant in the ODC1 gene in a girl with developmental delay, alopecia, and dysmorphic features. *Am J Med Genet A* 176: 2548 – 2553

Byers TL, Lakanen JR, Coward JK, Pegg AE (1994) The role of hypusine depletion in cytostasis induced by S-adenosyl-L-methionine decarboxylase inhibition: new evidence provided by 1-methylspermidine and 1,12-dimethylspermine. *Biochem J* 303: 363 – 368

© 2023 The Authors

Casero RA Jr, Murray Stewart T, Pegg AE (2018) Polyamine metabolism and cancer: treatments, challenges and opportunities. *Nat Rev Cancer* 18: 681 – 695

Cason AL, Ikeguchi Y, Skinner C, Wood TC, Holden KR, Lubs HA, Martinez F, Simensen RJ, Stevenson RE, Pegg AE *et al* (2003) X-linked spermine synthase gene (SMS) defect: the first polyamine deficiency syndrome. *Eur J Hum Genet* 11: 937 – 944

de Alencastro G, McCloskey DE, Kliemann SE, Maranduba CM, Pegg AE, Wang X, Bertola DR, Schwartz CE, Passos-Bueno MR, Sertie AL (2008) New SMS mutation leads to a striking reduction in spermine synthase protein function and a severe form of Snyder–Robinson X-linked recessive mental retardation syndrome. *J Med Genet* 45: 539 – 543

Glass JR, Gerner EW (1986) Polyamine-mediated turnover of ornithine decarboxylase in Chinese-hamster ovary cells. *Biochem J* 236: 351 – 357

Grigorenko NA, Khomutov AR, Keinänen TA, Järvinen A, Alhonen L, Jänne J, Vepsäläinen J (2007) Synthesis of novel optical isomers of α-methylpolyamines. *Tetrahedron* 63: 2257 – 2262

Holbert CE, Dunworth M, Foley JR, Dunston TT, Stewart TM, Casero RA (2020) Autophagy induction by exogenous polyamines is an artifact of bovine serum amine oxidase activity in culture serum. *J Biol Chem* 295: 9061 – 9068

Holbert CE, Cullen MT, Casero RA Jr, Stewart TM (2022) Polyamines in cancer: integrating organismal metabolism and antitumour immunity. *Nat Rev Cancer* 22: 467 – 480

Hyvonen MT, Keinanen TA, Cerrada-Gimenez M, Sinervirta R, Grigorenko N, Khomutov AR, Vepsalainen J, Alhonen L, Janne J (2007) Role of hypusinated eukaryotic translation initiation factor 5A in polyamine depletion-induced cytostasis. *J Biol Chem* 282: 34700 – 34706

Jarvinen A, Grigorenko N, Khomutov AR, Hyvonen MT, Uimari A, Vepsalainen J, Sinervirta R, Keinanen TA, Vujcic S, Alhonen L *et al* (2005) Metabolic stability of alpha-methylated polyamine derivatives and their use as substitutes for the natural polyamines. *J Biol Chem* 280: 6595 – 6601

Kabra PM, Lee HK, Lubich WP, Marton LJ (1986) Solid-phase extraction and determination of dansyl derivatives of unconjugated and acetylated polyamines by reversed-phase liquid chromatography: improved separation systems for polyamines in cerebrospinal fluid, urine and tissue. *J Chromatogr* 380: 19 – 32

Lakanen JR, Coward JK, Pegg AE (1992) alpha-Methyl polyamines: metabolically stable spermidine and spermine mimics capable of supporting growth in cells depleted of polyamines. *J Med Chem* 35: 724 – 734

Larcher L, Norris JW, Lejeune E, Buratti J, Mignot C, Garel C, Keren B, Schwartz CE, Whalen S (2019) The complete loss of function of the SMS gene results in a severe form of Snyder–Robinson syndrome. *Eur J Med Genet* 63: 103777

Lewis EC, Kraveka JM, Ferguson W, Eslin D, Brown VI, Bergendahl G, Roberts W, Wada RK, Oesterheld J, Mitchell D *et al* (2020) A subset analysis of a phase II trial evaluating the use of DFMO as maintenance therapy for high-risk neuroblastoma. *Int J Cancer* 147: 3152 – 3159

Li C, Brazill JM, Liu S, Bello C, Zhu Y, Morimoto M, Cascio L, Pauly R, Diaz-Perez Z, Malicdan MCV *et al* (2017) Spermine synthase deficiency causes lysosomal dysfunction and oxidative stress in models of Snyder–Robinson syndrome. *Nat Commun* 8: 1257

Liu R, Bi K, Jia Y, Wang Q, Yin R, Li Q (2012) Determination of polyamines in human plasma by high-performance liquid chromatography coupled with Q-TOF mass spectrometry. *J Mass Spectrom* 47: 1341 – 1346

LoGiudice N, Le L, Abuan I, Leizorek Y, Roberts SC (2018) Alpha-difluoromethylornithine, an irreversible inhibitor of polyamine biosynthesis, as a therapeutic strategy against hyperproliferative and infectious diseases. *Med Sci (Basel)* 6: 12

Mackintosh CA, Pegg AE (2000) Effect of spermine synthase deficiency on polyamine biosynthesis and content in mice and embryonic fibroblasts, and the sensitivity of fibroblasts to 1,3-bis-(2-chloroethyl)-N-nitrosourea. *Biochem J* 351: 439 – 447

Mamont PS, Danzin C, Wagner J, Siat M, Joder-Ohlenbusch AM, Claverie N (1982) Accumulation of decarboxylated S-adenosyl-L-methionine in mammalian cells as a consequence of the inhibition of putrescine biosynthesis. *Eur J Biochem* 123: 499 – 504

Meyskens FL Jr, Gerner EW (1999) Development of difluoromethylornithine (DFMO) as a chemoprevention agent. *Clin Cancer Res* 5: 945 – 951

Muñoz-Esparza NC, Latorre-Moratalla ML, Comas-Basté O, Toro-Funes N, Veciana-Nogués MT, Vidal-Carou MC (2019) Polyamines in food. *Front Nutr* 6: 108

Murray Stewart T, Khomutov M, Foley JR, Guo X, Holbert CE, Dunston TT, Schwartz CE, Gabrielson K, Khomutov A, Casero RA Jr (2020) (R,R)-1,12-Dimethylspermine can mitigate abnormal spermidine accumulation in Snyder–Robinson syndrome. *J Biol Chem* 295: 3247 – 3256

Murray-Stewart T, Dunworth M, Foley JR, Schwartz CE, Casero RA Jr (2018) Polyamine homeostasis in Snyder–Robinson syndrome. *Med Sci (Basel)* 6: E112

Pegg AE (1984) The role of polyamine depletion and accumulation of decarboxylated S-adenosylmethionine in the inhibition of growth of SV-3T3 cells treated with alpha-difluoromethylornithine. *Biochem J* 224: 29 – 38

Pegg AE (2009) Mammalian polyamine metabolism and function. *IUBMB Life* 61: 880 – 894

Pegg AE (2013) Toxicity of polyamines and their metabolic products. *Chem Res Toxicol* 26: 1782 – 1800

Pegg AE, Michael AJ (2010) Spermine synthase. *Cell Mol Life Sci* 67: 113 – 121

Pegg AE, Wang X, Schwartz CE, McCloskey DE (2011) Spermine synthase activity affects the content of decarboxylated S-adenosylmethionine. *Biochem J* 433: 139 – 144

Peng Y, Norris J, Schwartz C, Alexov E (2016) Revealing the effects of missense mutations causing Snyder–Robinson syndrome on the stability and dimerization of spermine synthase. *Int J Mol Sci* 17: 77

Peron A, Spaccini L, Norris J, Bova SM, Selicorni A, Weber G, Wood T, Schwartz CE, Mastrangelo M (2013) Snyder–Robinson syndrome: a novel nonsense mutation in spermine synthase and expansion of the phenotype. *Am J Med Genet A* 161A: 2316 – 2320

Rajasekaran S, Bupp CP, Leimanis-Laurens M, Shukla A, Russell C, Junewick J, Gleason E, VanSickle EA, Edgerly Y, Wittmann BM *et al* (2021) Repurposing eflornithine to treat a patient with a rare ODC1 gain-of-function variant disease. *Elife* 10: e67097

Regenass U, Mett H, Stanek J, Mueller M, Kramer D, Porter CW (1994) CGP 48664, a new S-adenosylmethionine decarboxylase inhibitor with broad spectrum antiproliferative and antitumor activity. *Cancer Res* 54: 3210 – 3217

Saulnier Sholler GL, Gerner EW, Bergendahl G, MacArthur RB, VanderWerff A, Ashikaga T, Bond JP, Ferguson W, Roberts W, Wada RK *et al* (2015) A phase I trial of DFMO targeting polyamine addiction in patients with relapsed/refractory neuroblastoma. *PLoS ONE* 10: e0127246

Schwartz CE, Peron A, Kutler MJ (1993) Snyder–Robinson syndrome. In *GeneReviews(®)*, Adam MP, Mirzaa GM, Pagon RA, Wallace SE, Bean LJH, Gripp KW, Amemiya A (eds). Seattle, WA: University of Washington

Schwartz CE, Wang X, Stevenson RE, Pegg AE (2011) Spermine synthase deficiency resulting in X-linked intellectual disability (Snyder–Robinson syndrome). *Methods Mol Biol* 720: 437 – 445

 © 2023 The Authors

Seiler N, Delcros JG, Moulinoux JP (1996) Polyamine transport in mammalian cells. An update. *Int J Biochem Cell Biol* 28: 843 – 861

Shantz LM, Holm I, Jänne OA, Pegg AE (1992) Regulation of S-adenosylmethionine decarboxylase activity by alterations in the intracellular polyamine content. *Biochem J* 288: 511 – 518

Snyder RD, Robinson A (1969) Recessive sex-linked mental retardation in the absence of other recognizable abnormalities. Report of a family. *Clin Pediatr (Phila)* 8: 669 – 674

Starks R, Kirby P, Ciliberto M, Hefti M (2018) Snyder–Robinson syndrome. *Autops Case Rep* 8: e2018031

Tabor CW, Rosenthal SM (1956) Pharmacology of spermine and spermidine; some effects on animals and bacteria. *J Pharmacol Exp Ther* 116: 139 – 155

Tao X, Zhu Y, Diaz-Perez Z, Yu SH, Foley JR, Stewart TM, Casero RA Jr, Steet R, Zhai RG (2022) Phenylbutyrate modulates polyamine acetylase and ameliorates Snyder–Robinson syndrome in a *Drosophila* model and patient cells. *JCI Insight* 7: e158457

Til HP, Falke HE, Prinsen MK, Willems MI (1997) Acute and subacute toxicity of tyramine, spermidine, spermine, putrescine and cadaverine in rats. *Food Chem Toxicol* 35: 337 – 348

VanSickle EA, Michael J, Bachmann AS, Rajasekaran S, Prokop JW, Kuzniecky R, Hofstede FC, Steindl K, Rauch A, Lipson MH *et al* (2021) Expanding the phenotype: four new cases and hope for treatment in Bachmann–Bupp syndrome. *Am J Med Genet A* 185: 3485 – 3493

Wang X, Levic S, Gratton MA, Doyle KJ, Yamoah EN, Pegg AE (2009) Spermine synthase deficiency leads to deafness and a profound sensitivity to alpha-difluoromethylornithine. *J Biol Chem* 284: 930 – 937

Wu H, Min J, Zeng H, McCloskey DE, Ikeguchi Y, Loppnau P, Michael AJ, Pegg AE, Plotnikov AN (2008) Crystal structure of human spermine synthase: implications of substrate binding and catalytic mechanism. *J Biol Chem* 283: 16135 – 16146

Zhang Z, Teng S, Wang L, Schwartz CE, Alexov E (2010) Computational analysis of missense mutations causing Snyder–Robinson syndrome. *Hum Mutat* 31: 1043 – 1049

License: This is an open access article under the terms of the Creative Commons Attribution License, which permits use, distribution and reproduction in any medium, provided the original work is properly cited.

Expanded View Figures

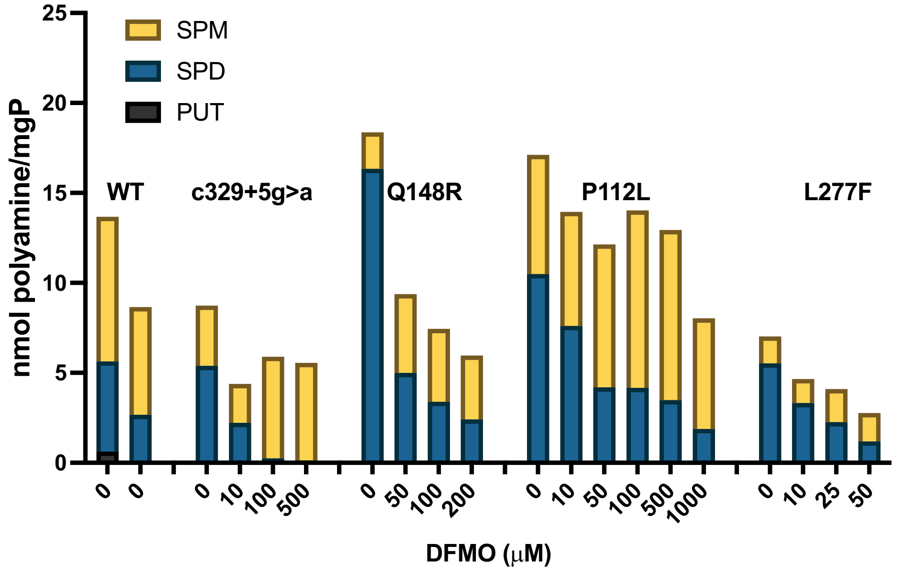

Figure EV1. Effect of DFMO on SPD and SPM pools in SRS patient fibroblast cell lines.

Fibroblasts with hypomorphic mutations of SMS (c.329+5, Q148R, P112L, and L277F) were treated for 96 h with increasing doses of DFMO and analyzed by HPLC for intracellular polyamine levels. A dose-dependent reduction in SPD is observed concurrent with increased SPM concentrations. Data represent the means of duplicate determinations and is presented as nmol polyamine per mg total protein in the lysate. Stacking of individual polyamine levels allows visualization of total polyamine concentration at baseline and with treatment. Cell lines from two different WT donors are included for comparison.

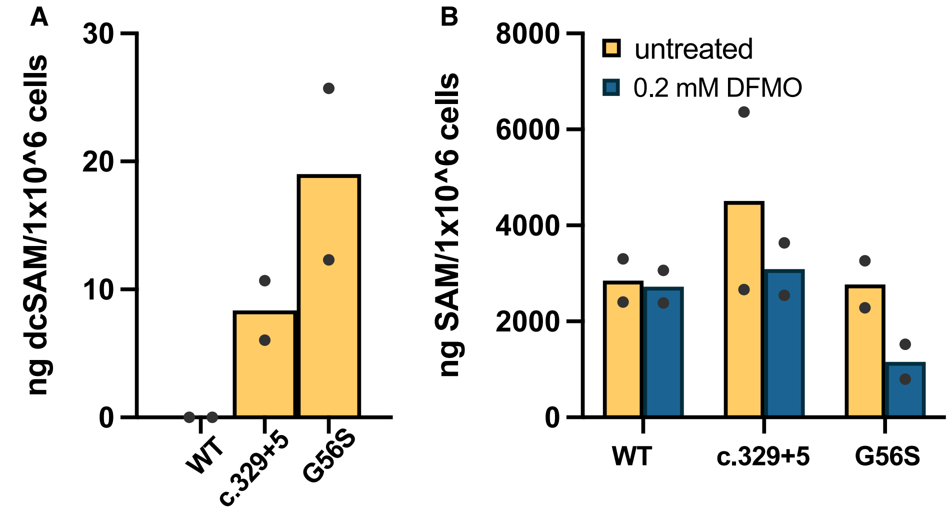

Figure EV2. Concentrations of dcSAM and SAM in lymphoblastoid cell lines.

A Baseline concentrations of dcSAM in SRS lymphoblasts compared to wildtype (WT) were determined by LCMS/MS. Levels were below the limit of detection in WT cells but were elevated in SRS cells, with the highest accumulation in cells from the more severely affected SMS^{G56S} variant.

B WT and SRS lymphoblasts were treated 96 h with 0.2 mM DFMO. SAM levels were quantified by LCMS/MS and normalized by cell number.

Data information: values are plotted from independent experiments with column height indicating the means.

 © 2023 The Authors

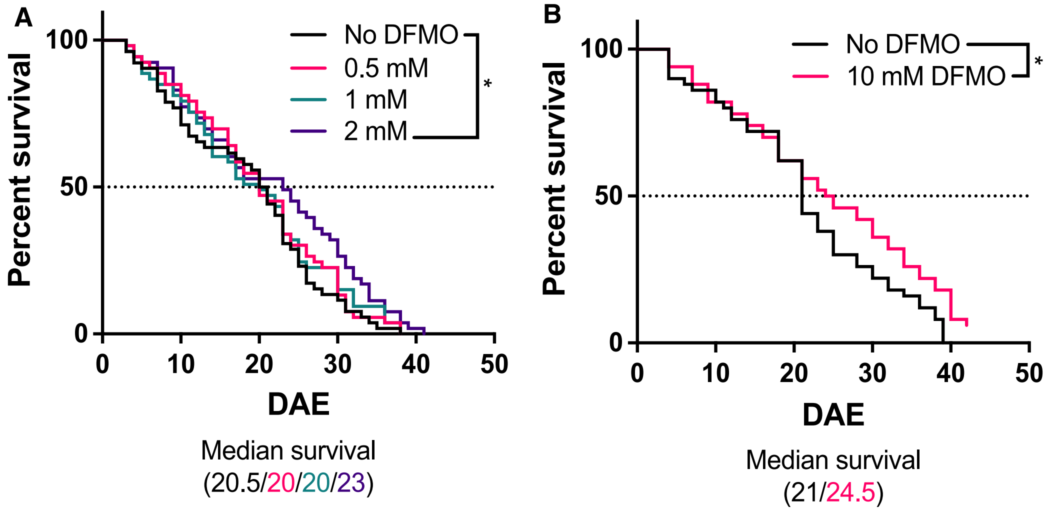

Figure EV3. DFMO extends lifespan in female *dSms*$^{-/-}$ Drosophila.

The indicated concentration of DFMO was administered in the feed and survival was observed.

A *n* = 52, 50, 53, and 53 in the 0, 0.5, 1, and 2-mM DFMO treatment groups, respectively.

B *n* = 50 and 53 in the 0 and 10-mM DFMO treatment groups, respectively.

Data information: The resulting survival curves were compared using the log-rank (Mantel–Cox) test. (WT vs. 2 mM DFMO, $P = 0.0312$; WT vs. 10 mM DFMO, $P = 0.038$). DAE, days after eclosure.
