# Supplementary material for: Difluoromethylornithine rebalances aberrant polyamine ratios in Snyder–Robinson syndrome
Source: EMBO Mol Med. 2023 Sep 13;15(11):e17833. doi: 10.15252/emmm.202317833 (PMC10630878; doi:10.15252/emmm.202317833)
Supplement: Supplementary file 7 [file EMMM-15-e17833-s005.pdf]

EMBO
Molecular Medicine

Difluoromethylornithine rebalances aberrant polyamine ratios in Snyder-Robinson syndrome

Jackson Foley, Cassandra Holbert, Maxim Khomutov, Noushin Rastkari, Xianzun Tao, Alex Khomutov, Grace Zhai, Robert A. Casero Jr. and Tracy Stewart
DOI: 10.15252/emmm.202317833

Corresponding author(s): Tracy Stewart (tmurray2@jhmi.edu)

Review Timeline:

Submission Date:	10th Apr 23
Editorial Decision:	16th May 23
Revision Received:	5th Jul 23
Editorial Decision:	10th Aug 23
Revision Received:	18th Aug 23
Accepted:	30th Aug 23

Editor: Lise Roth

Transaction Report:

(Note: With the exception of the correction of typographical or spelling errors that could be a source of ambiguity, letters and reports are not edited. Depending on transfer agreements, referee reports obtained elsewhere may or may not be included in this compilation. Referee reports are anonymous unless the Referee chooses to sign their reports.)

16th May 2023

Dear Dr. Stewart,

Thank you for the submission of your manuscript to EMBO Molecular Medicine, and please accept my apologies for the delay in getting back to you as one reviewer had to be withdrawn and replaced. We have now received feedback from the three reviewers who agreed to evaluate your manuscript. As you will see from the reports below, the reviewers acknowledge the interest of the study and are overall supporting publication of your work pending appropriate revisions.

Addressing the reviewers' concerns in full will be necessary for further considering the manuscript in our journal, and acceptance of the manuscript will entail a second round of review. EMBO Molecular Medicine encourages a single round of revision only and therefore, acceptance or rejection of the manuscript will depend on the completeness of your responses included in the next, final version of the manuscript. For this reason, and to save you from any frustrations in the end, I would strongly advise against returning an incomplete revision.

If you would like to discuss further the points raised by the referees, I am available to do so via email or video. Let me know if you are interested in this option.

Revised manuscripts should be submitted within three months of a request for revision; they will otherwise be treated as new submissions, except under exceptional circumstances in which a short extension is obtained from the editor.

When submitting your revised manuscript, please carefully review the instructions that follow below. We perform an initial quality control of all revised manuscripts before re-review; failure to include requested items will delay the evaluation of your revision.

We require:

1) A .docx formatted version of the manuscript text (including legends for main figures, EV figures and tables). Please make sure that the changes are highlighted to be clearly visible.

2) Individual production quality figure files as .eps, .tif, .jpg (one file per figure). For guidance, download the 'Figure Guide PDF' (https://www.embopress.org/page/journal/17574684/authorguide#figureformat).

3) At EMBO Press we ask authors to provide source data for the main figures. Our source data coordinator will contact you to discuss which figure panels we would need source data for and will also provide you with helpful tips on how to upload and organize the files.

4) A .docx formatted letter INCLUDING the reviewers' reports and your detailed point-by-point responses to their comments. As part of the EMBO Press transparent editorial process, the point-by-point response is part of the Review Process File (RPF), which will be published alongside your paper.

5) A complete author checklist, which you can download from our author guidelines (https://www.embopress.org/page/journal/17574684/authorguide#submissionofrevisions). Please insert information in the checklist that is also reflected in the manuscript. The completed author checklist will also be part of the RPF.

6) It is mandatory to include a 'Data Availability' section after the Materials and Methods. Before submitting your revision, primary datasets produced in this study need to be deposited in an appropriate public database, and the accession numbers and database listed under 'Data Availability'. Please remember to provide a reviewer password if the datasets are not yet public (see https://www.embopress.org/page/journal/17574684/authorguide#dataavailability).
Note that the Data Availability Section is restricted to new primary data that are part of this study.

7) For data quantification: please specify the name of the statistical test used to generate error bars and P values, the number (n) of independent experiments (specify technical or biological replicates) underlying each data point and the test used to calculate p-values in each figure legend. The figure legends should contain a basic description of n, P and the test applied. Graphs must include a description of the bars and the error bars (s.d., s.e.m.). Please provide exact p values.

8) Our journal encourages inclusion of *data citations in the reference list* to directly cite datasets that were re-used and obtained from public databases. Data citations in the article text are distinct from normal bibliographical citations and should directly link to the database records from which the data can be accessed. In the main text, data citations are formatted as follows: "Data ref: Smith et al, 2001" or "Data ref: NCBI Sequence Read Archive PRJNA342805, 2017". In the Reference list, data citations must be labeled with "[DATASET]". A data reference must provide the database name, accession number/identifiers and a resolvable link to the landing page from which the data can be accessed at the end of the reference. Further instructions are available at .

9) We replaced Supplementary Information with Expanded View (EV) Figures and Tables that are collapsible/expandable online. A maximum of 5 EV Figures can be typeset. EV Figures should be cited as 'Figure EV1, Figure EV2" etc... in the text and their respective legends should be included in the main text after the legends of regular figures.
- For the figures that you do NOT wish to display as Expanded View figures, they should be bundled together with their legends in a single PDF file called *Appendix*, which should start with a short Table of Content. Appendix figures should be referred to in the main text as: "Appendix Figure S1, Appendix Figure S2" etc.
- Additional Tables/Datasets should be labeled and referred to as Table EV1, Dataset EV1, etc. Legends have to be provided in a separate tab in case of .xls files. Alternatively, the legend can be supplied as a separate text file (README) and zipped together with the Table/Dataset file.
See detailed instructions here:

10) For more information: There is space at the end of each article to list relevant web links for further consultation by our readers. Could you identify some relevant ones and provide such information as well? Some examples are patient associations, relevant databases, OMIM/proteins/genes links, author's websites, etc...

11) Author contributions: CRediT has replaced the traditional author contributions section because it offers a systematic machine readable author contributions format that allows for more effective research assessment. Please remove the Authors Contributions from the manuscript and use the free text boxes beneath each contributing author's name in our system to add specific details on the author's contribution. More information is available in our guide to authors.

12) Disclosure statement and competing interests: We updated our journal's competing interests policy in January 2022 and request authors to consider both actual and perceived competing interests. Please review the policy https://www.embopress.org/competing-interests and update your competing interests if necessary.

13) Every published paper now includes a 'Synopsis' to further enhance discoverability. Synopses are displayed on the journal webpage and are freely accessible to all readers. They include a short stand first (maximum of 300 characters, including space) as well as 2-5 one-sentences bullet points that summarizes the paper. Please write the bullet points to summarize the key NEW findings. They should be designed to be complementary to the abstract - i.e. not repeat the same text. We encourage inclusion of key acronyms and quantitative information (maximum of 30 words / bullet point). Please use the passive voice. Please attach these in a separate file or send them by email, we will incorporate them accordingly.

Please also suggest a striking image or visual abstract to illustrate your article as a PNG file 550 px wide x 300-600 px high.

14) As part of the EMBO Publications transparent editorial process initiative (see our Editorial at http://embomolmed.embopress.org/content/2/9/329), EMBO Molecular Medicine will publish online a Review Process File (RPF) to accompany accepted manuscripts.
In the event of acceptance, this file will be published in conjunction with your paper and will include the anonymous referee reports, your point-by-point response and all pertinent correspondence relating to the manuscript. Let us know whether you agree with the publication of the RPF and as here, if you want to remove or not any figures from it prior to publication.
Please note that the Authors checklist will be published at the end of the RPF.

EMBO Molecular Medicine has a "scooping protection" policy, whereby similar findings that are published by others during review or revision are not a criterion for rejection. Should you decide to submit a revised version, I do ask that you get in touch after three months if you have not completed it, to update us on the status.

I look forward to receiving your revised manuscript.

With kind regards,

Lise Roth

Lise Roth, PhD
Senior Editor
EMBO Molecular Medicine

***** Reviewer's comments *****

Referee #2 (Comments on Novelty/Model System for Author):

Murray Stewart and co-workers used novel model systems to test their hypothesis, including human cells derived from SRS patients and a novel Drosophila SRS model characterized by reduced lifespan.

Referee #2 (Remarks for Author):

General comments

The manuscript by Murray Stewart et al reports a study that is an outstanding example of translational biomedical research. The paper addresses an unmet medical need in children with Snyder Robinson Syndrome (SRS), which is the lack of any currently effective pharmacotherapies. The authors build on previous studies implicating a loss of activity of the polyamine biosynthetic enzyme spermine synthase (SMS) in SRS, and others indicating that an imbalance in the relative amounts of the polyamines spermidine and spermine is associated with disease severity in SRS patients. The authors hypothesized that rebalancing the relative amounts of these polyamines could have a therapeutic benefit in these patients. Murray Stewart and co-workers used novel model systems to test their hypothesis, including human cells derived from SRS patients and a novel Drosophila SRS model characterized by reduced lifespan.

The authors recognized that most SRS patients are hypomorphic for germline SMS alterations. They found an expected decrease in pool sizes of putrescine, the diamine precursor of spermidine, and spermidine in SRS cells treated with difluoromethylornithine (DFMO), an enzyme-activated inhibitor of ornithine decarboxylase, but also found evidence of increased spermine synthesis in SRS cells with some residual SMS activity. This finding is consistent with other studies indicating that polyamine biosynthesis is itself highly regulated by a series of feedback mechanisms. They show that SRS cells retain the normal mechanism of regulating the production of the propyl amine precursor (dcSAM) by AMD1, but demonstrate that this mechanism only leads to spermine formation in SRS cells retaining some SMS activity.

The authors recognize that tissue polyamines can be derived from several processes including uptake from dietary or intestinal luminal sources. They use an interesting spermine mimetic, 1,12-Me2-spermine, to augment mechanistic studies indicating that cellular transport mechanisms involving uptake from outside to inside of cells is functional in SRS cells. The spermine mimetic is interesting in that it is resistant to catabolism by serum amine oxidases that might degrade it in patients. In cell culture experiments employing an inhibitor of amine oxidases, both spermine and the spermine mimetic (with or without the amine oxidase inhibitor) restored growth defects and corrected the relative amounts of spermidine and spermine in SRS cells.

Finally, the authors demonstrate that DFMO increases the lifespan of flies in the Drosophila model system, from 21 to 35 days, providing evidence of a therapeutic benefit for DFMO, and potentially the spermine mimetic, in a novel model of this disease.

Specific comments

1. See last line of abstract - The authors should consider the potential inclusion of a genetic marker capable of distinguishing hypomorphic genotypes from total loss of spermine synthase as a potential companion diagnostic for therapies like DFMO and/or the spermine mimetic. They note that "nearly all SRS patient mutations are hypomorphic..." Given the report by Pegg and co-workers that Gy mice, which lack SMS activity, are extremely sensitive to DFMO treatment, such a companion diagnostic could provide important patient safety data in any future clinical trials of this strategy.

2. Last para Intro - The fact that DFMO has been approved by regulatory agencies for other indications may be of limited relevance in this case. Here, the authors are considering chronic administration at low doses (see page 8 line 19 in manuscript) of both DFMO and/or the mimetic. Prior approvals have been for acute IV delivery of high doses of DFMO (sleeping sickness) or topical delivery (Vaniqa). Both these prior approvals are quite different from those contemplated for patients with SRS. Of more relevance will be ongoing studies involving chronic administration of lower doses, like those used to treat children with neuroblastoma.

Referee #3 (Remarks for Author):

This well-designed and well-written manuscript describes the potential use of DFMO in the treatment of patients with Snyder Robinson syndrome (SRS). Since most SRS patients have hypomorphic mutations of the SMS gene causing reduced SMS activity and reduced spermine levels, DFMO can rebalance the spermidine:spermine ratios in these patients by increasing production of dcSAM to facilitate spermine synthesis and by increasing cellular import of polyamines. There are a few points that should be addressed before publication:

1. Fig. 2A: What are the concentrations of DFMO used to treat the SRS patient-derived lymphoblastoid cells? Also, correct the spelling of lymphoblastoid in the figure title.
2. Since the effect of DFMO on the spermidine:spermine ratio is variable depending on the extent to which the mutation affects SMS activity, is there a possibility that lowering the ratio to where there is so little spermidine accumulated (as shown in Fig. S1 with c329+5g>a cells treated with 100 µM DFMO) would cause pathology too? Since spermidine is required for hypusination of eIF5A, how may DFMO affect activation of eIF5A?
3. Fig. 4A: in SMS-WT cells, what causes the decrease in the spermidine:spermine ratio - DFMO? This should be indicated.
4. Since DFMO cannot rebalance the spermidine:spermine ratio in cells with a complete loss of SMS activity, it is not clear how the Drosophila model with SMS knocked out is a good model for hypomorphic mutations of SMS in humans, nor the mechanism by which DFMO extends their lifespan. Please discuss. A mouse model with a hypomorphic mutation of SMS would be ideal for future preclinical studies.

Referee #4 (Remarks for Author):

In Snyder-Robinson syndrome, an incurable disease for which no fundamental treatment exists, the SPD/SPM ratio is increased as a result of the accumulation of SPD, the precursor of SPM, in addition to a decrease in the amount of SPM due to partial dysfunction of spermine synthase. The SPD/SPM ratio tends to be higher the more severe the symptoms of Snyder-Robinson syndrome. The authors carried out this study with the idea that the disease can be treated by reducing the elevated SPD/SPM ratio in patients with Snyder-Robinson syndrome. In a further in vivo study, using a fly model of Snyder-Robinson syndrome, it was shown that oral administration of DMFO increased the life span shortened by the disease.
The study is of great interest, but there are some major problems with the organization of the manuscript and some additional experiments are needed to integrate the results of the in intro and in vivo studies.

1. To integrate the fly experiments with your in vitro experiments up to that point, the following additional experiments should be added.
(a) Test the respective polyamine levels in the body of the flies.
(b) Analyse the changes in expression of polyamine-related enzyme genes (odc, srm, sms, amd1) in the fly body following DFMO administration.
(c) Analyse the effects of Spm alone and simultaneous administration of Spm and DFMO on the lifespan of flies.

2. Please prepare a Table showing genotype and characteristics, spermidine synthase activity and first published literature for the cells isolated from Snyder-Robinson syndrome patients and used in this study, and introduce them in the introduction.

3. The Legend for Fig. 2 and Fig. 4 describes the experimental results. The legend should contain detailed information about the experimental conditions and the axes of the graphs, while the experimental results should be integrated in the text. Please revise.

4. There is a citation to Fig. 3D in the text, but Fig. 3D does not exist in this manuscript. Either delete the citation in the text or add Fig. 3D. (p. 5, "included during treatment (Fig. 3B-D).")

5. Fig. 4C is present in Figures, but a citation to fig. 4C is missing in the text of this manuscript; please explain the results while citing fig. 4C, or delete fig. 4C.

6. In the Discussion, please specifically cite Figs for the results of this study. It should be cited in at least the following three places, but also in other areas where it is possible to cite it.
(a) While the extent of induction was similar between WT cells and the G56S variant, induction in the c.329+5 variant significantly exceeded either of these.
(b) Consequently, cells with this variant have measurable SMS activity that maintains a SPD/SPM ratio approximately double that of WT controls (our study and (5)).
(c) resulting in a ratio approximately 6-fold greater than controls (our study and (10))

7. Please explain the results more carefully, by mentioning data marked with Asterisk in the figures (What you have shown to be statistically significant differences) in the text.

8. Please provide statistical treatment for the bottom panel of Fig. 2A and indicate significant differences for data described as different in the text.

9. Two results for the SMSWT group for the same conditions are shown in Fig. 2A and Fig. 4A respectively. Please explain the reason, or delete one of them.

Minor points
Abstract

"Mechanistic studies"
Change to "In vitro studies".

Results
P. 4
Consistent with DFMO-mediated inhibition of biosynthesis, SPD was reduced in a dose-dependent manner;.
Insufficient data to describe "in a dose-dependent manner", as there is no statistically significant difference between DFMO concentrations of 0.1 mM and 0.5 mM (Fig. 2E).

P. 5
"These results indicate that the most common SRS-associated mutations, all of which are hypomorphic, increase resistance to the growth inhibitory effects of DFMO. Additionally, as extracellular sources of SPM include the diet and microbiota, growth inhibition by DFMO is not anticipated to be a treatment limitation in SRS."
Please move this sentence to Discussion. Also cite the appropriate literature on the provision of polyamines derived from the diet and microbiota. Furthermore, in the introduction the author described that food-derived spermine does not alter the Spd/Spm ratio, which seems contradictory at first glance. Please explain this more carefully.

Figure legend for Fig. 2
Please specify in the legend what the abbreviations NT and WT refer to.

***** Reviewer's comments *****

Referee #2 (Comments on Novelty/Model System for Author):

Murray Stewart and co-workers used novel model systems to test their hypothesis, including human cells derived from SRS patients and a novel Drosophila SRS model characterized by reduced lifespan.

Referee #2 (Remarks for Author):

General comments

The manuscript by Murray Stewart et al reports a study that is an outstanding example of translational biomedical research. The paper addresses an unmet medical need in children with Snyder Robinson Syndrome (SRS), which is the lack of any currently effective pharmacotherapies. The authors build on previous studies implicating a loss of activity of the polyamine biosynthetic enzyme spermine synthase (SMS) in SRS, and others indicating that an imbalance in the relative amounts of the polyamines spermidine and spermine is associated with disease severity in SRS patients. The authors hypothesized that rebalancing the relative amounts of these polyamines could have a therapeutic benefit in these patients. Murray Stewart and co-workers used novel model systems to test their hypothesis, including human cells derived from SRS patients and a novel Drosophila SRS model characterized by reduced lifespan.

The authors recognized that most SRS patients are hypomorphic for germline SMS alterations. They found an expected decrease in pool sizes of putrescine, the diamine precursor of spermidine, and spermidine in SRS cells treated with difluoromethylornithine (DFMO), an enzyme-activated inhibitor of ornithine decarboxylase, but also found evidence of increased spermine synthesis in SRS cells with some residual SMS activity. This finding is consistent with other studies indicating that polyamine biosynthesis is itself highly regulated by a series of feedback mechanisms. They show that SRS cells retain the normal mechanism of regulating the production of the propyl amine precursor (dcSAM) by AMD1, but demonstrate that this mechanism only leads to spermine formation in SRS cells retaining some SMS activity.

The authors recognize that tissue polyamines can be derived from several processes including uptake from dietary or intestinal luminal sources. They use an interesting spermine mimetic, 1,12-Me2-spermine, to augment mechanistic studies indicating that cellular transport mechanisms involving uptake from outside to inside of cells is functional in SRS cells. The spermine mimetic is interesting in that it is resistant to catabolism by serum amine oxidases that might degrade it in patients. In cell culture experiments employing an inhibitor of amine oxidases, both spermine and the spermine mimetic (with or without the amine oxidase inhibitor) restored growth defects and corrected the relative amounts of spermidine and spermine in SRS cells.

Finally, the authors demonstrate that DFMO increases the lifespan of flies in the Drosophila model system, from 21 to 35 days, providing evidence of a therapeutic benefit for DFMO, and potentially the spermine mimetic, in a novel model of this disease.

Specific comments

1. See last line of abstract - The authors should consider the potential inclusion of a genetic marker

capable of distinguishing hypomorphic genotypes from total loss of spermine synthase as a potential companion diagnostic for therapies like DFMO and/or the spermine mimetic. They note that "nearly all SRS patient mutations are hypomorphic..." Given the report by Pegg and co-workers that Gy mice, which lack SMS activity, are extremely sensitive to DFMO treatment, such a companion diagnostic could provide important patient safety data in any future clinical trials of this strategy.
Thank you for this suggestion. We have included the following in the Discussion:

"These results also suggest that clinical use of DFMO to treat SRS patients may need to be tailored to the individual depending on the amount of residual SMS activity or SPD/SPM ratio. Indeed, in cell lines with the c.329+5 g>a variant (**Fig 2A, S1**), exposure to DFMO ultimately depleted SPD pools, which may also be detrimental. However, we anticipate the *in vivo* situation to be less responsive in this regard due to compensatory uptake of extracellular PUT and/or SPD. Future studies in relevant mammalian animal models should clarify this potential. Regardless, the results of these studies indicate that a functional readout of SMS activity, such as SPD/SPM ratio, should be an important consideration in DFMO clinical trial design."

"However, an early mouse model that completely lacked the chromosomal region including the *Sms* gene was extremely sensitive to DFMO, which had detrimental effects (16). Whether this resulted from the lack of Sms activity or an alternative function is unclear, but caution is warranted when considering patient inclusion criteria for clinical trial design."

2. Last para Intro - The fact that DFMO has been approved by regulatory agencies for other indications may be of limited relevance in this case. Here, the authors are considering chronic administration at low doses (see page 8 line 19 in manuscript) of both DFMO and/or the mimetic. Prior approvals have been for acute IV delivery of high doses of DFMO (sleeping sickness) or topical delivery (Vaniqa). Both these prior approvals are quite different from those contemplated for patients with SRS. Of more relevance will be ongoing studies involving chronic administration of lower doses, like those used to treat children with neuroblastoma. We agree and have now emphasized the chronic administration of DFMO, such as that in NB, by adding the following sentence to the last paragraph of the Introduction:

"Notably, both BABS and neuroblastoma patients receive multiple oral doses of DFMO daily on a continuing basis, representing patient populations and dosing schedules similar to those anticipated in an SRS clinical trial."

Referee #3 (Remarks for Author):

This well-designed and well-written manuscript describes the potential use of DFMO in the treatment of patients with Snyder Robinson syndrome (SRS). Since most SRS patients have hypomorphic mutations of the SMS gene causing reduced SMS activity and reduced spermine levels, DFMO can rebalance the spermidine:spermine ratios in these patients by increasing production of dcSAM to facilitate spermine synthesis and by increasing cellular import of polyamines. There are a few points that should be addressed before publication:

1. Fig. 2A: What are the concentrations of DFMO used to treat the SRS patient-derived lymphoblastoid cells? The DFMO concentrations are noted below the bottom panel. This has now been clarified in the figure legend as follows:

"Cells were treated with increasing concentrations of DFMO as indicated below the bottom panel."

Also, correct the spelling of lymphoblastoid in the figure title. - done

2. Since the effect of DFMO on the spermidine:spermine ratio is variable depending on the extent to which the mutation affects SMS activity, is there a possibility that lowering the ratio to where there is so little spermidine accumulated (as shown in Fig. S1 with c329+5g>a cells treated with 100 µM DFMO) would cause pathology too? Since spermidine is required for hypusination of eIF5A, how may DFMO affect activation of eIF5A? Thank you for the reminder to include a discussion of this differential response and what it might mean for patients. Between the reduction in SMS activity and the compensatory uptake of polyamines that DFMO causes, it is unlikely that SPD will be depleted in the organismal setting in the dramatic manner observed in our cell lines in culture (in the absence of exogenous polyamines). We anticipate that cells will import enough PUT/SPD to maintain these polyamines at homeostatic levels. Future studies in animal models will be needed to fully address this question. We have included the following in the Discussion:

"These results also suggest that clinical use of DFMO to treat SRS patients may need to be tailored to the individual depending on the amount of residual SMS activity or SPD/SPM ratio. Indeed, in cell lines with the c.329+5 g>a variant (**Fig 2A, S1**), exposure to DFMO ultimately depleted SPD pools, which may also be detrimental. However, we anticipate the *in vivo* situation to be less responsive in this regard due to compensatory uptake of extracellular PUT and/or SPD. Future studies in a relevant mammalian animal model should clarify this potential. Regardless, the results of these studies will be important considerations for future clinical trial design."

3. Fig. 4A: in SMS-WT cells, what causes the decrease in the spermidine:spermine ratio - DFMO? This should be indicated.
Thank you for noting this. The WT results are from 2 different donors. We have included the following in the figure legend:

"Untreated lymphoblast lines from 2 different SMS^{WT} healthy donors are included for comparison of polyamine levels and SPD/SPM ratio."

4. Since DFMO cannot rebalance the spermidine:spermine ratio in cells with a complete loss of SMS activity, it is not clear how the Drosophila model with SMS knocked out is a good model for hypomorphic mutations of SMS in humans, nor the mechanism by which DFMO extends their lifespan. Please discuss. We agree completely that the fly model is not an ideal model, but it is currently the only system readily available with a defined phenotype amenable to attenuation. As our primary goal with this study was to define the mechanism-of-action of DFMO in SRS patient cells, we chose to use the fly model to expand the study to the organismal level and demonstrate an effect beyond that at the biochemical/molecular level. We have now included the following sentence in the Discussion to clarify that SPM is a component of the fly medium, suggesting that DFMO may improve SPM availability in support of lifespan extension:

"Additionally, the standard media used for *Drosophila* studies includes measurable SPM (approximately 4 nmol/mg), suggesting its enhanced uptake by the $dSms^{-/-}$ flies may be responsible for the extended lifespan in response to DFMO."

A mouse model with a hypomorphic mutation of SMS would be ideal for future preclinical studies.
We agree, and further studies are planned in a newly available mouse SRS model, once baseline phenotyping studies have been completed and a phenotype relevant to the human population has been identified. We have included the following sentence in the Discussion to indicate the status of the mouse studies:

"A mouse model of SRS, which harbors the clinically relevant Sms^{G56S} mutation, recently became available (The Jackson Laboratory, strain #033707). Our plans for future studies include determining the

effects of DFMO, SPM, and Me_2SPM on these mice; however, a patient-relevant phenotype must first be identified."

Referee #4 (Remarks for Author):

In Snyder-Robinson syndrome, an incurable disease for which no fundamental treatment exists, the SPD/SPM ratio is increased as a result of the accumulation of SPD, the precursor of SPM, in addition to a decrease in the amount of SPM due to partial dysfunction of spermine synthase. The SPD/SPM ratio tends to be higher the more severe the symptoms of Snyder-Robinson syndrome. The authors carried out this study with the idea that the disease can be treated by reducing the elevated SPD/SPM ratio in patients with Snyder-Robinson syndrome. In a further in vivo study, using a fly model of Snyder-Robinson syndrome, it was shown that oral administration of DMFO increased the life span shortened by the disease.
The study is of great interest, but there are some major problems with the organization of the manuscript and some additional experiments are needed to integrate the results of the in intro and in vivo studies.

1. To integrate the fly experiments with your in vitro experiments up to that point, the following additional experiments should be added.
(a) Test the respective polyamine levels in the body of the flies. This is a good suggestion and was in our original plan. Unfortunately, technical issues prohibited the collection of this data and our system has since been out of commission. However, we would like to emphasize that the choice to include the fly model was made with the goal of demonstrating an effect at the organismal level. As the biochemical MOA has been deciphered in human patient cells, we do not believe that the lack of *Drosophila* biochemistry diminishes the study. The SRS fly model has some major limitations (it is a complete knockout vs. hypomorph, *dSms* is autosomal vs. X-linked, and the fly polyamine pathway may differ from that in mammals), but it is the only readily available model with a defined, reversible phenotype. Thus, we used it to show a phenotypic benefit beyond the change in biochemistry observed in patient cells, but respectfully do not believe that further molecular studies in the fly model will add value to the manuscript.
(b) Analyse the changes in expression of polyamine-related enzyme genes (odc, srm, sms, amd1) in the fly body following DFMO administration. Due to the limitations described in 1a, we do not believe these experiments will add value to our study. Additionally, most polyamine-related genes, including those mentioned, are majorly regulated at the post-transcriptional/translational level, so measuring gene expression provides little indication of true metabolic activity.
(c) Analyse the effects of Spm alone and simultaneous administration of Spm and DFMO on the lifespan of flies. This is an important point we did not clarify. We did not supplement the fly growth medium with SPM, as we had determined it was already present in the ingredients. So, the conditions suggested are the same as those performed. This has been clarified in the Discussion as follows:

"Notably, the standard media used for *Drosophila* studies includes measurable SPM (approximately 4 nmol/mg), suggesting its enhanced uptake by the *dSms*$^{-/-}$ flies may be responsible for the extended lifespan in response to DFMO."

2. Please prepare a Table showing genotype and characteristics, spermidine synthase activity and first published literature for the cells isolated from Snyder-Robinson syndrome patients and used in this study, and introduce them in the introduction.
The following text with corresponding Table has been added in the Introduction. Note that although we

like the idea of including the patient characteristics, this requires much space and was recently provided in another publication, which is now emphasized for the reader. Also, we chose to include the ratio as a functional readout of SMS activity, as an actual activity assay is not currently in use.

"A summary of patient cell lines with ratios included in this study can be found in **Table 1.** Additionally, a detailed collection of the clinical features of the individual patients was recently published by Larcher and colleagues (14)."

Table 1 – Variants and ratios of SRS patient-derived cell lines in the current study.

**DNA variant**	**Protein**	**SPD/SPM ratio** means ± s.e.m. (n ≥ 2)	**Reference**
**WT**	WT	1.75 ± 0.18 (L)<br>0.54 ± 0.07 (f)	
**c.166g>a**	G56S	13.68 ± 0.78 (L)	(de Alencastro *et al.*, 2008)
**c.329+5g>a**	Truncated, novel protein with some WT	7.39 ± 1.20 (L)<br>1.50 ± 0.29 (f)	(Arena *et al.*, 1996; Cason *et al.*, 2003; Snyder & Robinson, 1969)
**c.335c>t**	P112L	1.74 ± 0.08 (f)	(Peng *et al.*, 2016)
**c.443a>g**	Q148R	9.43 ± 0.69 (f)	(Albert *et al.*, 2015)
**c.449t>c**	I150T	2.94 ± 0.58 (f)	(Zhang *et al.*, 2010)
**c.831g>t**	L277F	4.75 ± 0.83 (f)	(Starks *et al.*, 2018)
**c.906-909del**	M303K*fs3	N/A[1] (f)	(Larcher *et al.*, 2019)

[1] Ratio of N/A is due to complete lack of SPM; WT, wildtype; (L), lymphoblast; (f), fibroblast

3. The Legend for Fig. 2 and Fig. 4 describes the experimental results. The legend should contain detailed information about the experimental conditions and the axes of the graphs, while the experimental results should be integrated in the text. Please revise.

We have revised the figure legends according to the EMBO Molecular Medicine Author Guidelines.

4. There is a citation to Fig. 3D in the text, but Fig. 3D does not exist in this manuscript. Either delete the citation in the text or add Fig. 3D. (p. 5, "included during treatment (Fig. 3B-D).")
Thank you for catching this. The "D" has been changed to "C".

5. Fig. 4C is present in Figures, but a citation to fig. 4C is missing in the text of this manuscript; please explain the results while citing fig. 4C, or delete fig. 4C.
Thank you. Fig 4C has now been cited.

6. In the Discussion, please specifically cite Figs for the results of this study. It should be cited in at least the following three places, but also in other areas where it is possible to cite it.
(a) While the extent of induction was similar between WT cells and the G56S variant, induction in the c.329+5 variant significantly exceeded either of these.

(b) Consequently, cells with this variant have measurable SMS activity that maintains a SPD/SPM ratio approximately double that of WT controls (our study and (5)).
(c) resulting in a ratio approximately 6-fold greater than controls (our study and (10))
References to figures have now been added throughout the Discussion.

7. Please explain the results more carefully, by mentioning data marked with Asterisk in the figures (What you have shown to be statistically significant differences) in the text. We have carefully reviewed the Results and added text in cases of ambiguity.

8. Please provide statistical treatment for the bottom panel of Fig. 2A and indicate significant differences for data described as different in the text. Statistically significant differences are indicated in figures in accordance with EMBO's guidelines, which advise against providing statistics for studies with n = 2.

9. Two results for the SMSWT group for the same conditions are shown in Fig. 2A and Fig. 4A respectively. Please explain the reason, or delete one of them.
Thank you for noting this. The WT results are from 2 different donors. We have included the following in the figure legend:

"Untreated lymphoblast lines from 2 different *SMS*^{WT} healthy donors are included for comparison of polyamine levels and SPD/SPM ratio."

Minor points
Abstract
"Mechanistic studies"
Change to "In vitro studies". We have added "in vitro"

Results
P. 4
Consistent with DFMO-mediated inhibition of biosynthesis, SPD was reduced in a dose-dependent manner;.
Insufficient data to describe "in a dose-dependent manner", as there is no statistically significant difference between DFMO concentrations of 0.1 mM and 0.5 mM (Fig. 2E).

"in a dose-dependent manner" has been removed

P. 5
"These results indicate that the most common SRS-associated mutations, all of which are hypomorphic, increase resistance to the growth inhibitory effects of DFMO. Additionally, as extracellular sources of SPM include the diet and microbiota, growth inhibition by DFMO is not anticipated to be a treatment limitation in SRS."
Please move this sentence to Discussion. Also cite the appropriate literature on the provision of polyamines derived from the diet and microbiota. Done

Furthermore, in the introduction the author described that food-derived spermine does not alter the Spd/Spm ratio, which seems contradictory at first glance. Please explain this more carefully. Food-derived spermine seems to have limited effect on its own – this is the problem we hope to negate with DFMO. One possible explanation is that the cell type necessary for uptake and entry into systemic circulation has downregulated polyamine transport, unlike the fibroblasts and lymphoblasts we can easily study in culture. These types of studies will benefit from an appropriate mouse model. DFMO should stimulate uptake in all cell types, as well as stimulate the conversion of SPD to SPM. We have added the following in the Introduction:

“However, the fact remains that SPM is a common dietary component that fails to rescue the SRS phenotype *in vivo*, suggesting that improvements to SPM bioavailability may enhance efficacy.”

Figure legend for Fig. 2

Please specify in the legend what the abbreviations NT and WT refer to. - done

10th Aug 2023

Dear Dr. Stewart,

Thank you for the submission of your revised manuscript to EMBO Molecular Medicine, and please accept my apologies for the delay in getting back to you in this busy time of the year. We have now received the reports from the 2 referees who re-reviewed your manuscript. As mentioned in a previous correspondence and as you will see below, while referee #3 is supportive of publication, referee #4 regrets that concerns raised during the initial review have not been addressed.
Based on the explanations you provided and on the technical limitations mentioned, and after discussion with my colleagues, we agree that it would not be productive to wait for these limitations to be overcome, and I am therefore pleased to inform you that I will be able to accept your manuscript once the following editorial points will be addressed:

1/ Please provide a detailed response to referee #4's remaining concerns.

2/ Main manuscript text:
- Please address the queries from our data editors in the related manuscript file. Please accept previous changes and only keep in track changes mode any new modification.
- Please rename 'Experimental Procedures' 'Materials and methods'.
- Please move the Data Availability section to the end of the Materials and Methods.
- Acknowledgements: the information provided in this section should be the same as provided in the submission system. Currently, Commonwealth Foundation for Cancer Research, Chan-Zuckerberg Initiative have not been entered in the submission system.

3/ Figures:
- please make sure all figures/figure panels are referenced in the text. A callout is currently missing for Fig. 5A.
- please remove error bars in figure with n=2.

4/ Thank you for providing a nice synopsis image. Could you please send it as a jpg/png/tiff version as 550 pixels wide x 200-400 high?

5/ As part of the EMBO Publications transparent editorial process initiative (see our Editorial at http://embomolmed.embopress.org/content/2/9/329), EMBO Molecular Medicine will publish online a Review Process File (RPF) to accompany accepted manuscripts.
This file will be published in conjunction with your paper and will include the anonymous referee reports, your point-by-point response and all pertinent correspondence relating to the manuscript. Let us know whether you agree with the publication of the RPF and as here, if you want to remove or not any figures from it prior to publication.
Please note that the Authors checklist will be published at the end of the RPF.

I look forward to receiving your revised manuscript.

Yours sincerely,

Lise Roth

Lise Roth, PhD
Senior Editor
EMBO Molecular Medicine

***** Reviewer's comments *****

Referee #3 (Remarks for Author):

The authors have adequately addressed all the concerns of this reviewer.

Referee #4 (Remarks for Author):

The authors fully corrected the deficiencies in the manuscript, however, they rejected all three additional experiments with Drosophila that I proposed to conduct. I consider the first two experiments, other than the addition of Spm to the diet, to be essential for publication in the EMBO Molecular Medicine. I have therefore made the above decision.

***** Reviewer's comments *****

Referee #3 (Remarks for Author):

The authors have adequately addressed all the concerns of this reviewer.

Thank you for your positive review of our manuscript and suggestions for improvement.

Referee #4 (Remarks for Author):

The authors fully corrected the deficiencies in the manuscript, however, they rejected all three additional experiments with Drosophila that I proposed to conduct. I consider the first two experiments, other than the addition of Spm to the diet, to be essential for publication in the EMBO Molecular Medicine. I have therefore made the above decision.

Thank you for your time reviewing our manuscript. In previous studies, polyamine levels in flies were analyzed using our dedicated HPLC system (for our studies and other members of the polyamine community), and we attempted to measure those samples exposed to DFMO. However, our system required repair with parts we were no longer able to source. We currently remain without a functional HPLC, and as we generally serve as a source of this assay to the polyamine community, are unaware of an alternative. We expect to have a new system installed in the near future, however, it is unlikely we would have been able to repeat the lifespan studies and optimize the detection method on the new system within the resubmission window.

We regret that the fly polyamine data are currently unavailable and hope to follow-up on these studies in the future. The fact remains that the lifespan extension in Drosophila provides evidence of phenotype reversal at the organismal level, while we have already shown biochemical reversal in human patient cells. As there is ongoing progress in moving DFMO into SRS patients, we are eager for publication of our work in patient cells and believe the further characterization of Drosophila polyamine biochemistry to be outside the necessary scope of this project.

30th Aug 2023

Dear Dr. Stewart,

Thank you for submitting your revised files. I am pleased to inform you that your manuscript is accepted for publication and is now being sent to our publisher to be included in the next available issue of EMBO Molecular Medicine!

We would like to remind you that as part of the EMBO Publications transparent editorial process initiative, EMBO Molecular Medicine will publish a Review Process File online to accompany accepted manuscripts. If you do NOT want the file to be published or would like to exclude figures, please immediately inform the editorial office via e-mail.

Please read below for additional IMPORTANT information regarding your article, its publication and the production process.

Congratulations on your interesting work!

With kind regards,

Lise Roth

Lise Roth, Ph.D
Senior Editor
EMBO Molecular Medicine

Follow us on Twitter @EmboMolMed
Sign up for eTOCs at embopress.org/alertsfeeds

*** *** *** IMPORTANT INFORMATION *** *** ***

SPEED OF PUBLICATION
The journal aims for rapid publication of papers, using using the advance online publication "Early View" to expedite the process: A properly copy-edited and formatted version will be published as "Early View" after the proofs have been corrected. Please help the Editors and publisher avoid delays by providing e-mail address(es), telephone and fax numbers at which author(s) can be contacted.

Should you be planning a Press Release on your article, please get in contact with embomolmed@wiley.com as early as possible, in order to coordinate publication and release dates.

LICENSE AND PAYMENT:

All articles published in EMBO Molecular Medicine are fully open access: immediately and freely available to read, download and share.

EMBO Molecular Medicine charges an article processing charge (APC) to cover the publication costs. You, as the corresponding author for this manuscript, should have already received a quote with the article processing fee separately. Please let us know in case this quote has not been received.

Once your article is at Wiley for editorial production you will receive an email from Wiley's Author Services system, which will ask you to log in and will present you with the publication license form for completion. Within the same system the publication fee can be paid by credit card, an invoice, pro forma invoice or purchase order can be requested.

Payment of the publication charge and the signed Open Access Agreement form must be received before the article can be published online.

PROOFS

You will receive the proofs by e-mail approximately 2 weeks after all relevant files have been sent o our Production Office. Please return them within 48 hours and if there should be any problems, please contact the production office at embopressproduction@wiley.com.

Please inform us if there is likely to be any difficulty in reaching you at the above address at that time. Failure to meet our deadlines may result in a delay of publication.

All further communications concerning your paper proofs should quote reference number EMM-2023-17833-V3 and be directed to the production office at embopressproduction@wiley.com.

EMBO Press Author Checklist

Corresponding Author Name: Tracy Murray Stewart	
Journal Submitted to: EMBO Mol Med	
Manuscript Number: EMM-2023-17833	

USEFUL LINKS FOR COMPLETING THIS FORM
The EMBO Journal - Author Guidelines
EMBO Reports - Author Guidelines
Molecular Systems Biology - Author Guidelines
EMBO Molecular Medicine - Author Guidelines

Reporting Checklist for Life Science Articles (updated January
This checklist is adapted from Materials Design Analysis Reporting (MDAR) Checklist for Authors. MDAR establishes a minimum set of requirements in transparent reporting in the life sciences (see Statement of Task: 10.31222/osf.io/9sm4x). Please follow the journal's guidelines in preparing your manuscript.
Please note that a copy of this checklist will be published alongside your article.

Abridged guidelines for figures
1. Data
The data shown in figures should satisfy the following conditions:
- the data were obtained and processed according to the field's best practice and are presented to reflect the results of the experiments in an accurate and unbiased manner.
- ideally, figure panels should include only measurements that are directly comparable to each other and obtained with the same assay.
- plots include clearly labeled error bars for independent experiments and sample sizes. Unless justified, error bars should not be shown for technical
- if n<5, the individual data points from each experiment should be plotted. Any statistical test employed should be justified.
- Source Data should be included to report the data underlying figures according to the guidelines set out in the authorship guidelines on Data

2. Captions
Each figure caption should contain the following information, for each panel where they are relevant:
- a specification of the experimental system investigated (eg cell line, species name).
- the assay(s) and method(s) used to carry out the reported observations and measurements.
- an explicit mention of the biological and chemical entity(ies) that are being measured.
- an explicit mention of the biological and chemical entity(ies) that are altered/varied/perturbed in a controlled manner.
- the exact sample size (n) for each experimental group/condition, given as a number, not a range;
- a description of the sample collection allowing the reader to understand whether the samples represent technical or biological replicates (including how many animals, litters, cultures, etc.).
- a statement of how many times the experiment shown was independently replicated in the laboratory.
- definitions of statistical methods and measures:
  - common tests, such as t-test (please specify whether paired vs. unpaired), simple χ2 tests, Wilcoxon and Mann-Whitney tests, can be unambiguously identified by name only, but more complex techniques should be described in the methods section;
  - are tests one-sided or two-sided?
  - are there adjustments for multiple comparisons?
  - exact statistical test results, e.g., P values = x but not P values < x;
  - definition of 'center values' as median or average;
  - definition of error bars as s.d. or s.e.m.

Please complete ALL of the questions below.
Select "Not Applicable" only when the requested information is not relevant for your study.

Materials

Newly Created Materials	Information included in the manuscript?	In which section is the information available? (Reagents and Tools Table, Materials and Methods, Figures, Data Availability Section)
New materials and reagents need to be available; do any restrictions apply?	Not Applicable	

Antibodies	Information included in the manuscript?	In which section is the information available? (Reagents and Tools Table, Materials and Methods, Figures, Data Availability Section)
For **antibodies** provide the following information:<br>- Commercial antibodies: RRID (if possible) or supplier name, catalogue number and or/clone number<br>- Non-commercial: RRID or citation	Not Applicable	

DNA and RNA sequences	Information included in the manuscript?	In which section is the information available? (Reagents and Tools Table, Materials and Methods, Figures, Data Availability Section)
**Short novel DNA or RNA including primers, probes**: provide the sequences.	Not Applicable	

Cell materials	Information included in the manuscript?	In which section is the information available? (Reagents and Tools Table, Materials and Methods, Figures, Data Availability Section)
**Cell lines:** Provide species information, strain. Provide accession number in repository **OR** supplier name, catalog number, clone number, and/**OR** RRID.	Yes	Experimental procedures<br>Cell lines, culture conditions, and compounds and Table 1
**Primary cultures:** Provide species, strain, sex of origin, genetic modification status.	Yes	Experimental procedures<br>Cell lines, culture conditions, and compounds and Table 1
Report if the cell lines were recently **authenticated** (e.g., by STR profiling) and tested for mycoplasma contamination.	Yes	Experimental procedures<br>Cell lines, culture conditions, and compounds

Experimental animals	Information included in the manuscript?	In which section is the information available? (Reagents and Tools Table, Materials and Methods, Figures, Data Availability Section)
**Laboratory animals or Model organisms:** Provide species, strain, sex, age, genetic modification status. Provide accession number in repository **OR** supplier name, catalog number, clone number, **OR** RRID.	Yes	Experimental procedures<br>Drosophila studies and references therein
**Animal observed in or captured from the field:** Provide species, sex, and age where possible**.**	Not Applicable	
Please detail **housing and husbandry conditions**.	Yes	Experimental procedures<br>Drosophila studies and references therein

Plants and microbes	Information included in the manuscript?	In which section is the information available? (Reagents and Tools Table, Materials and Methods, Figures, Data Availability Section)
**Plants:** provide species and strain, ecotype and cultivar where relevant, unique accession number if available, and source (including location for collected wild specimens).	Not Applicable	
**Microbes:** provide species and strain, unique accession number if available, and source.	Not Applicable	

Human research participants	Information included in the manuscript?	In which section is the information available? (Reagents and Tools Table, Materials and Methods, Figures, Data Availability Section)
If collected and within the bounds of privacy constraints report on age, sex and gender or ethnicity for all study participants.	Not Applicable	

Core facilities	Information included in the manuscript?	In which section is the information available? (Reagents and Tools Table, Materials and Methods, Figures, Data Availability Section)
If your work benefited from core facilities, was their service mentioned in the acknowledgments section?	Yes	Acknowledgments

Design

Study protocol	Information included in the manuscript?	In which section is the information available? (Reagents and Tools Table, Materials and Methods, Figures, Data Availability Section)
If study protocol has been **pre-registered, provide DOI in the manuscript**. For clinical trials, provide the trial registration number **OR** cite DOI.	Not Applicable	
Report the **clinical trial registration number** (at ClinicalTrials.gov or equivalent), where applicable.	Not Applicable	

Laboratory protocol	Information included in the manuscript?	In which section is the information available? (Reagents and Tools Table, Materials and Methods, Figures, Data Availability Section)
Provide DOI OR other citation details if **external detailed step-by-step protocols** are available.	Not Applicable	

Experimental study design and statistics	Information included in the manuscript?	In which section is the information available? (Reagents and Tools Table, Materials and Methods, Figures, Data Availability Section)
Include a statement about **sample size** estimate even if no statistical methods were used.	Yes	Experimental procedures: Cell lines
Were any steps taken to minimize the effects of subjective bias when allocating animals/samples to treatment (e.g. **randomization procedure**)? If yes, have they been described?	Yes	Experimental procedures: Drosophila studies
Include a statement about **blinding** even if no blinding was done.	Yes	Experimental procedures: SAM and dcSAM quantification
Describe **inclusion/exclusion criteria** if samples or animals were excluded from the analysis. Were the criteria pre-established?<br><br>If sample or data points were omitted from analysis, report if this was due to attrition or intentional exclusion and provide justification.	Not Applicable	
For every figure, are **statistical tests** justified as appropriate? Do the data meet the assumptions of the tests (e.g., normal distribution)? Describe any methods used to assess it. Is there an estimate of variation within each group of data? Is the variance similar between the groups that are being statistically compared?	Yes	Experimental procedures: Statistical Analyses, Figure legends and EV Figure legends

Sample definition and in-laboratory replication	Information included in the manuscript?	In which section is the information available? (Reagents and Tools Table, Materials and Methods, Figures, Data Availability Section)
In the figure legends: state number of times the experiment was **replicated** in laboratory.	Yes	Figure legends and EV figure legends
In the figure legends: define whether data describe **technical or biological replicates**.	Yes	Figure legends and EV figure legends

Ethics

Ethics	Information included in the manuscript?	In which section is the information available? (Reagents and Tools Table, Materials and Methods, Figures, Data Availability Section)
Studies involving **human participants**: State details of **authority granting ethics approval** (IRB or equivalent committee(s), provide reference number for approval.	Not Applicable	
Studies involving **human participants**: Include a statement confirming that **informed consent** was obtained from all subjects and that the experiments conformed to the principles set out in the WMA Declaration of Helsinki and the Department of Health and Human Services Belmont Report.	Not Applicable	
Studies involving **human participants:** For publication of **patient photos**, include a statement confirming that consent to publish was obtained.	Not Applicable	
Studies involving experimental **animals**: State details of **authority granting ethics approval** (IRB or equivalent committee(s), provide reference number for approval. Include a statement of compliance with ethical regulations.	Not Applicable	
Studies involving **specimen and field samples:** State if relevant **permits** obtained, provide details of authority approving study; if none were required, explain why.	Not Applicable	

Dual Use Research of Concern (DURC)	Information included in the manuscript?	In which section is the information available? (Reagents and Tools Table, Materials and Methods, Figures, Data Availability Section)
Could your study fall under dual use research restrictions? Please check biosecurity documents and list of **select agents and toxins** (CDC): https://www.selectagents.gov/sat/list.htm	Not Applicable	
If you used a select agent, is the security level of the lab appropriate and reported in the manuscript?	Not Applicable	
If a study is subject to dual use research of concern regulations, is the name of the **authority granting approval and reference number** for the regulatory approval provided in the manuscript?	Not Applicable	

Reporting

The MDAR framework recommends adoption of discipline-specific guidelines, established and endorsed through community initiatives. Journals have their own policy about requiring specific guidelines and recommendations to complement MDAR.

Adherence to community standards	Information included in the manuscript?	In which section is the information available? (Reagents and Tools Table, Materials and Methods, Figures, Data Availability Section)
State if relevant guidelines or checklists (e.g., **ICMJE, MIBBI, ARRIVE, PRISMA**) have been followed or provided.	Yes	ICMJE guidelines for authorship - during submission
For **tumor marker prognostic studies,** we recommend that you follow the **REMARK** reporting guidelines (see link list at top right). See author guidelines, under 'Reporting Guidelines'. Please confirm you have followed these guidelines.	Not Applicable	
For **phase II and III randomized controlled trials**, please refer to the **CONSORT** flow diagram (see link list at top right) and submit the CONSORT checklist (see link list at top right) with your submission. See author guidelines, under 'Reporting Guidelines'. Please confirm you have submitted this list.	Not Applicable	

Data Availability

Data availability	Information included in the manuscript?	In which section is the information available? (Reagents and Tools Table, Materials and Methods, Figures, Data Availability Section)
Have **primary datasets** been deposited according to the journal's guidelines (see 'Data Deposition' section) and the respective accession numbers provided in the Data Availability Section?	Not Applicable	
Were **human clinical and genomic datasets** deposited in a public access-controlled repository in accordance to ethical obligations to the patients and to the applicable consent agreement?	Not Applicable	
Are **computational models** that are central and integral to a study available without restrictions in a machine-readable form? Were the relevant accession numbers or links provided?	Not Applicable	
If publicly available data were reused, provide the respective **data citations in the reference list**.	Not Applicable	
